# Maternal Inheritance of a Single Somatic Animal Cell Displayed by the Bacteriocyte in the Whitefly *Bemisia tabaci*

**DOI:** 10.1016/j.cub.2017.12.041

**Published:** 2018-02-05

**Authors:** Junbo Luan, Xuepeng Sun, Zhangjun Fei, Angela E. Douglas

**Affiliations:** 1 Department of Entomology, Cornell University, Ithaca, NY 14853, USA; 2 Boyce Thompson Institute, Ithaca, NY 14853, USA; 3 USDA-Agricultural Research Service, Robert W. Holley Center for Agriculture and Health, Ithaca, NY 14853, USA; 4 Department of Molecular Biology and Genetics, Cornell University, Ithaca, NY 14853, USA

**Keywords:** bacteriocyte, cellular immortality, maternal inheritance, microsatellite genotyping, symbiosis, telomeres, whiteflies

## Abstract

Bacteriocytes are insect cells harboring symbiotic bacteria that are required by their insect host and are transmitted vertically via the female ovary [1]. In most insect groups, the bacteria are released from the bacteriocytes and transferred to the ovary [2, 3], but in whiteflies, maternal bacteriocytes migrate to each egg [4, 5, 6], where they have been reported to lyse, releasing the symbionts [1]. To investigate bacteriocyte inheritance in whiteflies further, we applied microsatellite genotyping and genomic analysis to a genetically diverse population of *Bemisia tabaci*, and we observed the fate of the bacteriocyte in embryos. Surprisingly, the microsatellite profile of the bacteriocytes was uniform, and insect cross experiments demonstrated that the bacteriocytes have a stable genotype that differs from the genotype of the insect head (which lacks bacteriocytes). Comparative genomic analysis indicates that genomes of the bacteriocyte and whitefly head are distinct. Interestingly, the bacterioyte genome contains the canonical arthropod telomere repeats TTAGG, and the bacteriocytes express telomere maintenance genes that may underlie cellular immortality in animal cells [7]. Microscopy observations confirmed that a single bacteriocyte transmitted to each egg is retained and divides once just before egg hatch, yielding two bacteriocytes in the neonate insect. These data demonstrate the maternal inheritance of an absolutely required somatic insect cell, violating the developmental separation of germline and soma [8, 9]. Future investigation on the mechanism and phylogenetic distribution of maternally inherited bacteriocytes will shed light on the developmental origins and evolutionary diversification of bacteriocytes [10] and the processes underlying cellular immortality [11].

## Highlights

• Whitefly bacteriocytes have different microsatellite alleles from other somatic cells
• The bacteriocyte genotype is stable over three sexual generations of the insect
• Bacteriocyte genomes in different insects are genetically very similar
• The single bacteriocyte transmitted to the sexual egg persists through embryogenesis

## Graphical Abstract

**Figure:**
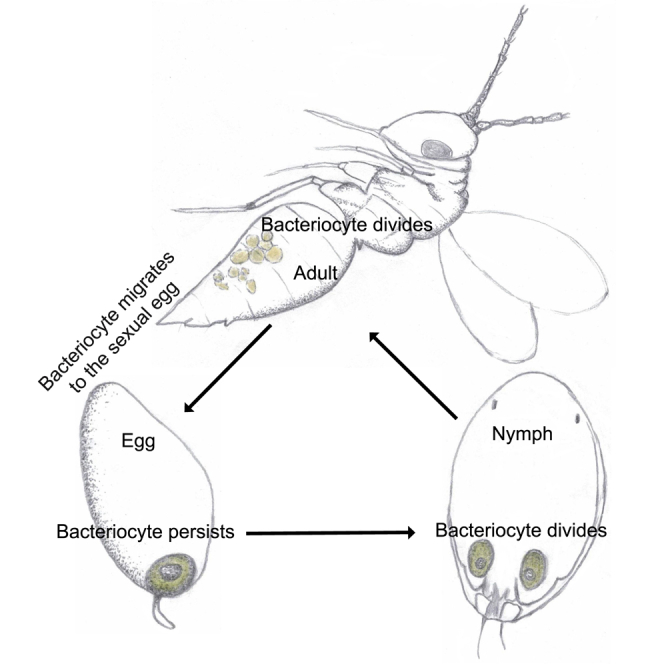

## Teaser

Luan et al. find that a cell bearing symbiotic bacteria is transferred to each sexual egg of whiteflies and retained through embryo development, leading to the maternal inheritance of a somatic cell with divergent genotype from other cells in the insect body. This mode of inheritance violates the germline/soma separation in animal development.

## Results

### Bacteriocytes Have the Same Microsatellite Alleles in the Whitefly Population

**Figure 1:**
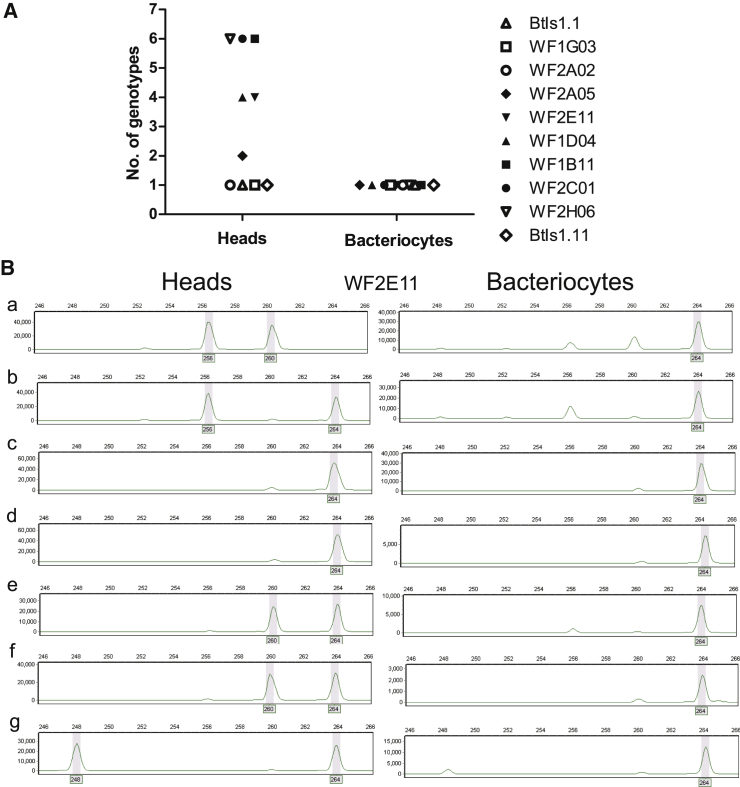
Genetic Variation of Bacteriocytes in the *Bemisia tabaci* Population The insects were adult females haphazardly selected from the routine insect culture. (A) Genetic variation of bacteriocytes and heads from different adult female whiteflies revealed by microsatellite profiles for ten markers. Two sets of ten insects were sampled, one for heads and the other for bacteriocytes. (B) Genetic variation of bacteriocytes and heads from the same female adult whiteflies represented by microsatellite profiles for the marker WF2E11. Each head and bacteriocyte sample was collected from each of seven insects (a–g). The gray shading denotes the allele identified for this sample. The minor peaks with the sizes ranging from 248 to 260 in bacteriocytes are likely contamination by DNA from other insect tissues, which cannot be excluded completely during bacteriocyte dissections of these very small insects. See also Table S1 and Data S1.

Our first experiment scored the genetic variation of whiteflies using 10 microsatellite markers (Table S1). Analysis of the heads (which have no bacteriocytes) of 10 female insects revealed polymorphisms in 6 of the 10 microsatellite loci (Figure 1A; Data S1A). In contrast, bacteriocytes dissected from a different haphazardly selected set of 10 female insects had a uniform microsatellite profile. To investigate the basis for this difference, we then determined the microsatellite profile from the head and bacteriocytes taken from the same insect. Across 7 replicate adult female insects, each of the five microsatellites tested yielded multiple genotypes for the heads but a single genotype for each microsatellite that was identical to that in the first analysis for the bacteriocytes (Figure 1B; Data S1B). Furthermore, one allele present in bacteriocytes was absent from the head samples and vice versa (Figure 1B: a, b, and e–g).

These data suggest that bacteriocytes in the whitefly population are genetically both more uniform than, and different from, other somatic tissues of the insects. Taken together with the microscopical evidence that a single whitefly bacteriocyte migrates from the body cavity to each unfertilized egg in the female ovary [1, 4], these data raise the possibility that the bacteriocytes may be maternally inherited.

### Bacteriocytes Have Stable Genotypes over Three Sexual Generations of the Insect

**Figure 2:**
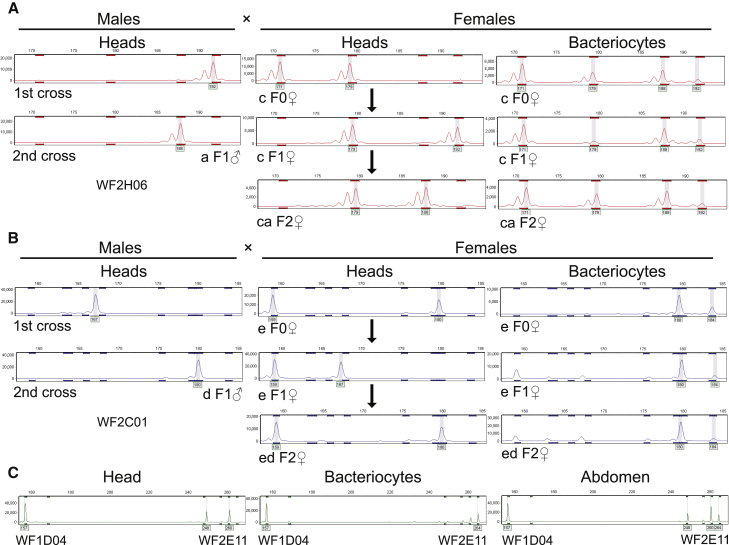
Genetic Variation of Bacteriocytes over Three Sexual Generations of *Bemisia tabaci* (A and B) Representative microsatellite profiles in male heads, female heads, and female bacteriocytes of adult whiteflies for the microsatellite marker WF2H06 in cross C (A) and for the microsatellite marker WF2C01 in cross F (B) over three generations. The bacteriocytes and heads were isolated from the same adult female whiteflies (data for all microsatellite loci are provided in Data S2). The arrows refer to generation of female progeny by crossing one female and one male. The gray shading denotes the allele identified for this sample. The minor peaks with the sizes ranging from 159 to 167 in (B) are likely contamination by other insect tissues (see legend to Figure 1) and peak stutter. (C) Microsatellite profiles for the markers WF1D04 and WF2E11 in the head, bacteriocytes, and abdomen of the same F2 adult whitefly in cross B. The gray shading denotes the allele identified for this sample. The minor peaks with the sizes ranging from 248 to 260 indicate possible contamination by other body tissue of insects during dissection and peak stutter. See also Tables S1 and S2 and Data S2.

To investigate whether bacteriocyte genotypes can be stably inherited over several generations, we designed 10 cross experiments using various combinations of whiteflies with diverse genotypes. Five polymorphic microsatellite loci were scored for female bacteriocytes, female heads, and male heads over three generations (Data S2). Because whiteflies are haplodiploid, we predicted that female offspring would have one maternal allele and one paternal allele, and this was observed for the head genotype in every female offspring tested in F1 and F2. In contrast, the bacteriocyte genotypes in the female offspring (F1 and F2) were identical to those in F0 female adult whiteflies (Figures 2A and 2B; Data S2). In some cases, the bacteriocyte and head alleles in a single insect were different (F1 in Figures 2A and 2B; Data S2). Furthermore, four alleles were detected in the bacteriocyte (but not head) samples with the microsatellite marker WF2H06 (Figure 2A), raising the possibility that the bacteriocyte nucleus may be polyploid.

To test for the possibility that the microsatellite primers amplified DNA from the maternally inherited bacterial symbionts, we conducted Sanger sequencing of PCR products for all five microsatellite loci in two of the cross experiments (cross-C and cross-F in Data S2) over three generations. All the products of microsatellite loci had 97%–100% of sequence identity to the whitefly genome and no detectable identity to the genome of either *Portiera* or *Hamiltonella*, the two endosymbiotic bacteria in the bacteriocytes (Table S2). These data demonstrate that the loci tested in the bacteriocyte samples are of insect origin.

We reasoned that, if the bacteriocytes (which reside in the insect abdomen) are the only cells that are somatically inherited, then the microsatellite profile of the insect abdomen should comprise both the alleles in the head and bacteriocyte. To test this prediction, we isolated the head, a subset of the bacteriocytes, and the abdomen with some remaining bacteriocytes from one female adult in the F2 offspring, and we tested the genotypes. In this individual, the head had two alleles and the bacteriocytes had one different allele for the microsatellite marker WF2E11. As expected, the abdomen including bacteriocytes contained all three alleles (Figure 2C).

### Bacteriocyte Genome Is Distinct from Whitefly Genome

The observation above indicates that the bacteriocyte genome could be different from the genome of other somatic cells in whiteflies. To investigate this possibility, genome resequencing was conducted on the bacteriocytes and head dissected from each of two adult female whiteflies selected at random from the population. We generated 10.5–31.1 Gb raw data for the four samples (two bacteriocyte samples and two head samples). After removing low-quality and adaptor sequences and collapsing duplicated reads, we obtained 3.5–8.3 Gb final cleaned sequences (mean coverage 5–12×; Table S3), which were used for variant calling with the *B. tabaci* MEAM1 genome as a reference [12]. A total of 513,556 variants were identified among these four samples, which included 453,208 SNPs (biallelic, 451,709; multiallelic, 1,499) and 60,348 small insertions or deletions (indels) (Table S3).

**Figure 3:**
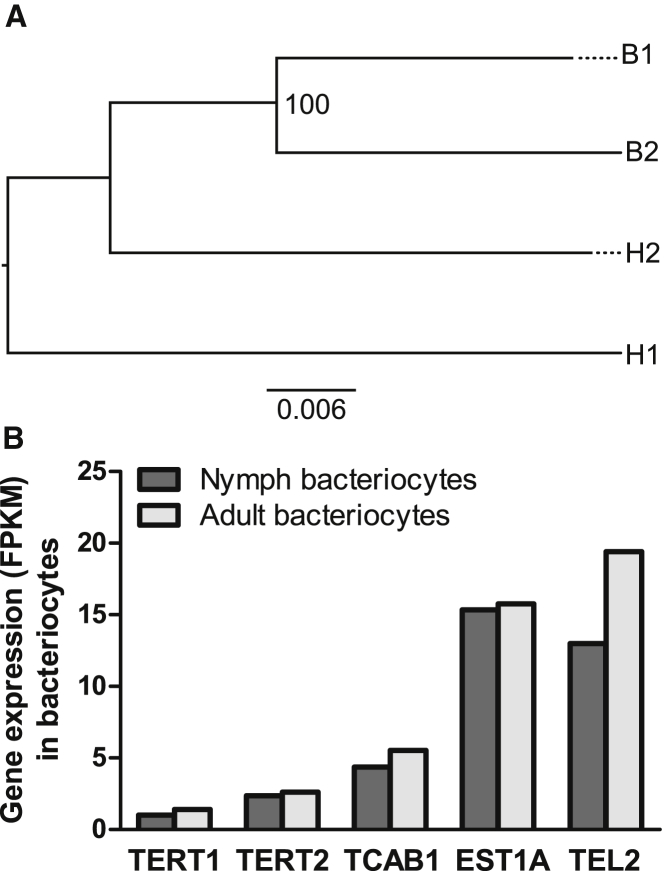
Bacteriocytes Have the Genome Distinct from Whitefly Genome Represented by Heads and Express Telomere Maintenance Genes (A) Genetic distance of bacteriocytes (B1 and B2) and head (H1 and H2) from two individuals shown by maximum likelihood phylogeny. B1 and H1 are from one individual whitefly and B2 and H2 are from another individual whitefly. The tree was constructed using homozygous SNPs and is mid-point rooted. The number on branch is the bootstrap value. (B) Expression of telomere maintenance genes in nymph bacteriocytes and adult bacteriocytes. The data were obtained by mapping raw reads of bacteriocyte transcriptome [4, 13] to the whitefly genome [12] and the FPKM value (fragments per kilobase of transcript per million fragments mapped) was calculated. See also Figure S1 and Tables S3 and S4.

To infer the genetic distance between the samples, we constructed a phylogenetic tree using all biallelic SNPs. The tree showed that the two head samples clustered together and separated from bacteriocyte samples (Figure S1). We reasoned that some of the heterozygous calls in bacteriocytes could be due to contamination of the dissected bacteriocytes by other tissues (see legend of Figure 1). We therefore reconstructed the phylogeny using variants comprising biallelic SNPs that are homozygous in every sample (1,775 sites). This tree indicated that the two bacteriocytes were closely related, and the two head samples were relatively more distant from each other than the bacteriocytes (Figure 3A). This observation is consistent with results from microsatellite analyses (Figures 1 and 2). In conclusion, the genomic evidence further confirms that bacteriocytes are not inherited in parallel with other somatic cells, as represented by the insect heads.

### Bacteriocytes Possess Canonical Arthropod Telomere Repeats of TTAGG and Express Telomere Maintenance Genes

In addition to being maternally inherited (as shown above), bacteriocytes are actively dividing cells, with an estimated 8–10 cell divisions over the lifespan of an individual insect [4]. We predicted that this remarkable cellular immortality is associated with the expression of telomerase, which stabilizes chromosomal telomeres in other immortal cells, including stem cells, germ cells, and many cancer cells; in normal soma cells, telomerase is not expressed, and these cells lose a portion of the telomere at each mitosis and eventually die [7, 14].

By analyzing the genome resequencing reads, we found that the bacteriocyte genome contained the canonical telomere repeat (TTAGG)_n_, which is the ancestral condition for insects and present in members of the order Hemiptera (which includes whiteflies) investigated to date [15, 16]. We also quantified the expression of genes with key functions in telomere maintenance, including those encoding the following: telomerase reverse transcriptase (TERT), which mediates the addition of nucleotides in a TTAGG sequence to telomeres; telomerase Cajal body protein 1 (TCAB1), required for telomere trafficking and synthesis in cancer cells; telomerase-binding protein EST1A; and telomere length regulation protein TEL2, using bacteriocyte transcriptome sequencing data of both nymphs and adults [4, 13]. All these genes were expressed in the bacteriocytes of both nymphs and adults of whiteflies (Figure 3B; Table S4). Taken together, these data suggest that the immortality of the bacteriocytes in *B. tabaci* may be underlain by the retention of the (TTAGG)_n_ telomere repeat and expression of telomere maintenance genes.

### Dynamics of Bacteriocytes and Associated Nuclei during Whitefly Embryogenesis

**Figure 4:**
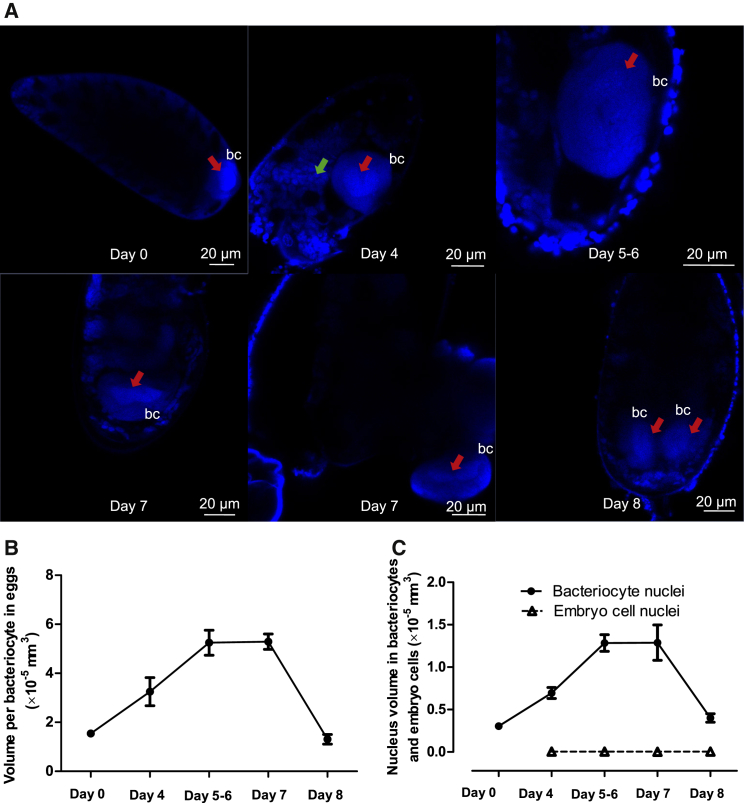
Dynamics of Bacteriocytes and Associated Nuclei during Whitefly Embryogenesis (A) Localization of bacteriocytes and associated nuclei in eggs at 0, 4, 5–6, 7, and 8 days post-oviposition, revealed by Hoechst 33342 staining of DNA. The bacterial symbionts that pack the cytoplasm of bacteriocytes are evident in the bacteriocyte periphery. bc, bacteriocyte; red arrow, bacteriocyte nucleus; green arrow, egg nucleus. (B) Bacteriocyte volume in eggs at 0, 4, 5–6, 7, and 8 days post-oviposition. Bacteriocyte volume was calculated from the diameter, assuming that the cell is a sphere, and it varied significantly with time (ANOVA: *F*_4,24_ = 25.06, p < 0.001). Data are represented as mean ± SEM (5 replicates). (C) Nucleus volume of the bacteriocytes (solid line) and embryo cells (broken line) in eggs at 0, 4, 5–6, 7, and 8 days post-oviposition. The volume of the bacteriocyte nuclei varied significantly with time (ANOVA: *F*_4,24_ = 18.57, p < 0.001), but the volume of nuclei of other cells in the embryos did not vary significantly (ANOVA: *F*_3,19_ = 3.98, p = 0.027). Data are represented as mean ± SEM (5 replicates).

To investigate the cellular basis of the maternal inheritance of whitefly bacteriocytes, we monitored the dynamics of bacteriocytes during whitefly embryogenesis (Figure 4). A single bacteriocyte was localized to the posterior region of newly deposited eggs at day 0 (1 hr post-oviposition). This bacteriocyte subsequently took up a medial location at day 4, and it increased progressively in size at 5–6 days post-oviposition. By day 7, the bacteriocyte returned to the posterior pole, prior to cell division. All embryos just prior to hatch (day 8; Figure 4A) and neonate nymphs [4] bore two bacteriocytes. The onset of bacteriocyte division between day 7 and day 8 was coincident with an approximate halving of the bacteriocyte volume (Figure 4B) and a significant reduction in size of the nucleus (Figure 4C). The mean volume of other nuclei in the embryo was both much smaller than that of bacteriocyte nuclei and did not vary significantly with developmental age (Figure 4C). Taken together, our microscopy observations confirm the genetic and molecular evidence that maternal bacteriocytes are inherited by the next insect generation.

## Discussion

According to conventional wisdom, the separation of the germline and soma is crucial for sustained cooperation among cells of animals and other multicellular organisms [9, 17, 18]. It is argued that, because all somatic cells are derived from the zygote nucleus, they are genetically identical and, therefore, have common genetic interests. The importance of the soma/germline separation is illustrated by the principal known exceptions: various cancer cells, whose immortality is deleterious to the individual. Although most cancers die with their host, there are rare instances of maternal inheritance of leukemia cells in humans [19], as well as lineages of contagious tumor cells in dogs and the Tasmanian devil *Sarcophilus harrisii* [20, 21].

Despite the predicted evolutionary conflicts, we provide multiple lines of evidence to support that the bacteriocytes in the whitefly *B. tabaci* are somatically inherited: the incongruent microsatellite alleles between bacteriocytes and somatic tissues (the insect head, which is bacteriocyte free), the strict inheritance of bacteriocyte alleles through sexual generations of the insect, the genomic differences between the bacteriocytes and head of individual insects, and the continuity of the bacteriocyte through embryo development. The fate of bacteriocytes in embryos of *B. tabaci* has not been investigated previously, and our results differ from an early microscopical study of symbiont transmission in another whitefly species, *Aleurodes proletella*, where multiple bacteriocytes transferred to each egg are reported to degrade in the embryo, followed by incorporation of the symbiotic bacteria into recently differentiated bacteriocytes of embryo origin [1]. Although the analysis of *A. proletella* was not supported by any genetic data, this likely difference points to two different fates of maternal bacteriocytes in embryos of different whitefly species. A parsimonious interpretation is that the somatic inheritance of bacteriocytes in *B. tabaci* may have evolved from the condition reported for *A. proletella*. This evolutionary transition may not be unique, and future studies focusing on the Coccoidea (hemipteran insects related to whiteflies) may be particularly fruitful [22]. In particular, an early study is suggestive of the somatic inheritance of bacteriocytes in the coccid *Puto* [1]. A different developmental arrangement occurs in the coccid *Pseudococcus* and diaspidid coccids, where the bacteriocyte is pentaploid, being derived anew in each insect generation from the fusion of the three polar bodies generated during oocyte development with the diploid nucleus of one embryo cell [23, 24, 25].

Further research is required to establish the antiquity of the bacteriocyte lineage in *B. tabaci*. As for asexual lineages in general, the bacteriocyte lineage may be evolutionarily short-lived as a result of accumulating deleterious mutations [26, 27]. Countering this process, however, is the very strong selection for bacteriocyte function. Whiteflies feed on plant phloem sap, which is grossly deficient in essential amino acids [28], and insect fitness is absolutely dependent on the sustained overproduction of essential amino acids by the symbiotic bacterium *Portiera* maintained within the bacteriocytes [13]. Because a single bacteriocyte is transmitted to each whitefly offspring, each individual bacteriocyte is exposed to selection at each insect generation. In this way, bacteriocytes with deleterious mutations are eliminated, and, potentially, mutations that specifically enhance symbiosis function (but are selectively neutral or deleterious in other somatic cells) may be favored. Candidates for positive selection in bacteriocytes are metabolism genes that are expressed out of their normal metabolic context to contribute to shared metabolic pathways with the bacterial symbiont [13]. Long-term persistence of the bacteriocyte lineage may also be promoted by genetic exchange (by mechanisms that are currently unknown), as occurs in some ancient animal lineages without canonical sex [29, 30].

Pertinent to the discussion of the antiquity of maternally inherited bacteriocytes in *B. tabaci* is the architecture of the bacteriocyte genome. The very large size of the bacteriocyte nucleus suggests that the bacteriocyte genome may be polyploid, likely through genome endoreduplication (i.e., multiple rounds of genome replication without cell division), as occurs commonly in other insect somatic cells, including bacteriocytes of other species [31, 32, 33]. Mutations in a persistently polyploid genome (possibly accompanied by genetic exchange, as proposed above) are predicted to lead to genetic diversification, but extensive genetic variation was not evident in our analysis of the microsatellite loci of the bacteriocytes. A priority for further research is to quantify the number of genome copies in each bacteriocyte nucleus and to estimate the scale of genetic variation among the genome copies. A related issue is the developmental origin of the bacteriocyte in *B. tabaci*. These cells may have evolved from the putative ancestral whitefly bacteriocyte (which develops *de novo* in each insect generation, as reported in *A. proletella*) by suppression of maternal bacteriocyte degradation in the embryo. Alternatively, the bacteriocytes in *B. tabaci* may have a distinctive developmental origin, possibly derived from a whitefly cell lineage that gained immortality through somatic mutation(s) and subsequently gained dramatically enhanced fitness by incorporating the obligate symbionts. Under either scenario, conflict between the bacteriocyte lineage and cells of sexual origin is suppressed by their mutual dependence on sustained bacterial function.

In summary, the maternal inheritance of the bacteriocyte in the whitefly *B. tabaci* represents a remarkable exception to the strict separation of the germline and soma, a central tenet of Weismann’s “doctrine of the continuity of the germline” [9]. Further investigation into the molecular and selective processes by which the whitefly bacteriocyte persists will provide new insights into the genetic basis of the individual in animals and the (im)mortality of cell lineages [11, 34], as well as contributing to our understanding of coevolved mutualisms between animals and their bacterial symbionts.

## STAR★Methods

### Key Resources Table

**Table:** 

REAGENT or RESOURCE	SOURCE	IDENTIFIER
**Chemicals, Peptides, and Recombinant Proteins**		

Multiplex PCR Plus Kit	QIAGEN	Cat# 206151
Nextera XT DNA library prep kit	Illumina	Cat# FC-131-1024
Bleach	Clorox	N/A
Paraformaldehyde	SIGMA-ALDRICH	P6148; CAS: 30525-89-4
Triton X-100	SIGMA-ALDRICH	X100; CAS: 9002-93-1
Hoechst 33342	Thermo Scientific	Cat# H3570; CAS: 23491-52-3

**Deposited Data**		

Genome of bacteriocyte sample B1	NCBI SRA	SRA: SRR6148276; https://www.ncbi.nlm.nih.gov/sra/?term=SRR6148276
Genome of bacteriocyte sample B2	NCBI SRA	SRA: SRR6148277; https://www.ncbi.nlm.nih.gov/sra/?term=SRR6148277
Genome of head sample H1	NCBI SRA	SRA: SRR6148278; https://www.ncbi.nlm.nih.gov/sra/?term=SRR6148278
Genome of head sample H2	NCBI SRA	SRA: SRR6148279; https://www.ncbi.nlm.nih.gov/sra/?term=SRR6148279
RNA-Seq data of bacteriocytes of nymph whiteflies	NCBI SRA [4],	SRA: SRR2001505; https://www.ncbi.nlm.nih.gov/sra/?term=SRR2001505
RNA-Seq data of bacteriocytes of adult whiteflies	NCBI SRA [13],	SRA: SRR1523521; https://www.ncbi.nlm.nih.gov/sra/?term=SRR1523521

**Experimental Models: Organisms/Strains**		

The whitefly *B. tabaci* MEAM1 culture (mtCOI GenBank accession number KM507785)	[4, 13]	N/A

**Software and Algorithms**		

Genemarker	SoftGenetics	http://www.softgenetics.com/GeneMarker.php
Trimmomatic v 0.35	[35]	http://www.usadellab.org/cms/?page=trimmomatic
BWA-MEM v 0.7.15-r1140	[36]	http://bio-bwa.sourceforge.net
Picard v 2.10.6	http://broadinstitute.github.io/picard/	https://github.com/broadinstitute/picard
SAMtools v 1.3	[37]	http://samtools.sourceforge.net
Freebayes v 0.9.21	[38]	https://github.com/ekg/freebayes
GATK HaplotypeCaller	[39]	https://software.broadinstitute.org/gatk
IQ-TREE v1.5.5	[40]	http://www.iqtree.org
SNPRelate	[41]	http://bioconductor.org/packages/release/bioc/html/SNPRelate.html
STAR	[42]	https://github.com/alexdobin/STAR
Excel	Microsoft	https://www.microsoft.com/en-us
GraphPad Prism 5	GraphPad Software	https://www.graphpad.com/scientific-software/prism

### Contact for Reagent and Resource Sharing

Further information and requests for resources and reagents should be directed to and will be fulfilled by the Lead Contact, Angela E. Douglas (aes326@cornell.edu).

### Experimental Model and Subject Details

The whitefly *B. tabaci* MEAM1 culture (mtCOI GenBank accession number KM507785) was obtained from poinsettia (*Euphorbia pulcherrima* Willd. Ex Klotzsch) in Ithaca, NY, USA in 1989. The culture was provided by Dr John Sanderson (Cornell University) to the authors in 2013, and has subsequently been maintained on dwarf cherry tomato (*Solanum lycopersicum* cv. Florida Lanai) in climate-controlled chambers at 27 ± 1°C with a 14 h light:10 h dark regime. The insects are maintained in large cages with at least 1,000 adults per cage, to maintain the genetic diversity.

### Method Details

#### Genetic variation of bacteriocytes in the whitefly population

To investigate genetic variation of bacteriocytes in a whitefly population, single pupae were cut from different leaves of multiple tomato plants in different cages and transferred to individual glass tubes (0.4 × 4cm) until they developed to adulthood. The bacteriocytes were dissected with fine pins from 10 replicate adult female whiteflies, each on a separate glass microscope slide at 40 × magnification, and then washed free of contaminating insect tissues with PBS (pH 7.4). The heads were cut off from the female adults of another ten whiteflies, respectively. For collection of each sample (female head, female bacteriocytes and male head), new pins, new slides and new tips were used to prevent cross-contamination of DNA. All the samples were immediately subjected to DNA extraction using the Nonidet-P40-based protocol. All instruments using for dissections and DNA extraction were pre-sterilized. Ten microsatellite loci developed for *B.tabaci* [43, 44] were amplified by PCR multiplexing with QIAGEN Multiplex PCR Plus Kit, using primers listed in Table S1. The PCR products were sent for fragment analysis. The alleles were analyzed using the software Genemarker (SoftGenetics LLC., USA) following the user manual. Finally, the microsatellite profiles of bacteriocytes and heads were compared.

#### Genetic variation of bacteriocytes over three generations in cross experiments

To examine genetic variation of bacteriocytes over three sexual generations, each newly-emerged unmated adult female and one newly-emerged unmated adult male (prepared as above) were released into a clip-cage that was secured to the abaxial surface of a tomato leaf at the 3-4 true-leaf stage. Females were allowed to oviposit for one week, and then the two adult insects from seven F0 mating pairs (the females were labeled as a-g) were collected for microsatellite determination of heads (both sexes) and bacteriocytes (females only: the numbers of bacteriocytes of adult males are extremely low, too few for microsatellite analysis), as described above, using 5 microsatellite markers as listed in Table S1. The leaf bearing the eggs (generation F1) produced by each F0 cross was excised from the plant and transferred to a 50 mL plastic tube as described previously [45]. Once the F1 insects emerged, they were collected into individual tubes, as for F0. The F1 female offspring were crossed with the F1 male offspring in 10 cross combinations to generate F2 insects as shown in Data S2. This process was repeated to determine the microsatellite profiles of the insects in the F1 crosses and F2. Finally, the microsatellite profiles of bacteriocytes and heads over the three generations were compared.

In parallel, the PCR products in cross experiments C and F over there generations were also Sanger sequenced to confirm the PCR products are from whiteflies or symbionts using primers for five microsatellite markers.

#### Library construction and genome resequencing

Two female adult whiteflies were randomly collected from different cages of the whitefly culture. Bacteriocytes were isolated from the individual female adult whiteflies, respectively, and washed with PBS. The heads were separated from the same two insects. All the samples were immediately subjected to DNA extraction using the Nonidet-P40-based protocol with the same precautions as described above to avoid contamination, including cross-contamination between samples. The total DNA was measured by Qubit^®^ 3.0 Fluorometer (Thermo Fisher Scientific Inc.). Illumina paired-end libraries were constructed using the Nextera^®^ xt DNA library prep kit, following the manufacturer’s instructions (Illumina, San Diego, CA, USA). These libraries were sequenced on the Illumina NextSeq 500 system with the paired-end mode and the read length of 76 bp.

#### Variant calling

The raw paired-end reads were trimmed for adapters and low quality bases using Trimmomatic v0.35 [35]. Cleaned read pairs were mapped to the whitefly *B. tabaci* MEAM1 genome [12] using BWA-MEM v 0.7.15-r1140 [36], with –M option to mark split alignments as secondary. Alignments with mapping quality ≥ 20 were retained and duplicated reads were marked using Picard v 2.10.6 (http://broadinstitute.github.io/picard/). Variants were pre-called using SAMtools v1.3 [37] and Freebayes v0.9.21 [38], respectively with recommended commands: “samtools mpileup -uf genome_file bam_file | bcftools call -Ov -mv” and “freebayes -F 0.2 -C 2 -p 2 -b bam_file -f genome_file.” Variants called by the two programs were filtered separately using the criteria: 1) variant quality ≥ 30; 2) depth ≥ 10 & ≤ 300; 3) no significant strand bias; and 4) no missing genotypes. The overlapped sites from the two filtered calls were extracted and used for base quality score recalibration in the final variant calling using GATK HaplotypeCaller [39]. The GATK analysis was performed following the online Best Practices protocol with default parameters (https://software.broadinstitute.org/gatk/best-practices/). Variants called by GATK were filtered using bcftools (http://samtools.github.io/bcftools/) with the criteria: 1) variant quality ≥ 30; 2) minimal depth for each sample ≥ 2; 3) at least 20 bp away from an InDel; and 4) no missing genotypes. Phylogenic analyses using biallelic SNPs were performed with IQ-TREE v1.5.5 [40]. Best nucleotide substitution model was chosen by model test function in IQ-TREE, and ascertainment bias correction (ASC) was applied for likelihood calculation on SNP data. Finally, the transversion model (TVMe+ASC) and Jukes-Cantor type model (MK+ASC) were used on phylogeny inference for homozygous SNPs and whole SNPs, respectively. The program was run with 1000 bootstrap replicates and both trees were mid-point rooted. We also used SNPRelate [41] to perform relatedness analysis using identity-by-descent methods, and this generated consistent tree topology.

#### Expression of telomere maintenance genes in bacteriocytes

Raw RNA-Seq data of bacteriocytes of nymph whiteflies [4] (Acc# SRR1523521) and adult whiteflies [13] (Acc# SRR2001505) were downloaded from NCBI Sequence Read Archive (SRA). Raw RNA-Seq reads were processed using Trimmomatic [35] to remove adaptor and low quality sequences. The cleaned reads were aligned to the whitefly *B. tabaci* MEAM1 genome [12] using STAR [42]. Following alignments, raw counts for telomere maintenance genes were derived and normalized to fragments per kilobase of exon model per million mapped fragments (FPKM).

#### Observation of bacteriocytes and nuclei during whitefly embryogenesis

Approximately 30 female adults of whiteflies were released into each of 50 clip-cages attached to the leaves and allowed to lay eggs for 1 h and then discarded. Eggs were collected at day 0 (1 h post oviposition), day 4, day 5, day 6, day 7 and day 8 after deposition. Eggs deposited at day 0 were dechorionated by 60% Clorox bleach (3.6% hypochlorite) in PBS for 5 min and then wash with PBS twice, fixed by 4% paraformaldehyde (PFA) at room temperature for 1 h, and then permeabilized with 0.1% Triton X-100 in PBS for 1.5 h. The samples were incubated with Hoechst 33342 (10 μg ml^-1^ in PBS, Thermo Scientific) at room temperature for 20 min. After the dechorionation treatment, the nuclei in the embryos at late embryogenesis did not stain well. So, we designed a pin-puncture approach to promote permeation of the reagent and dye into eggs. Punctured eggs at days 4-8 after deposition and bacteriocytes dissected from eggs at day 7 after deposition were fixed in 4% PFA at 4°C overnight and then permeabilized with 0.1% Triton X-100 in PBS at room temperature for 2 h. The samples were incubated with Hoechst 33342 (10 μg ml^-1^ in PBS, Thermo Scientific) in PBS overnight at 4°C. Images were collected and analyzed on a Zeiss LSM 700 confocal microscope. The diameters of the bacteriocytes in eggs and of the nuclei of bacteriocytes and embryo cells were determined by software ZEN for the Zeiss LSM 700 confocal microscope, using five eggs at each stage of embryogenesis and five embryo nuclei per egg. Bacteriocyte and nucleus volume was calculated as 4/3πr^3^ as described previously [4].

### Quantification and Statistical Analysis

#### Analysis of volume of bacteriocytes and nuclei

The volume of five embryo nuclei in each egg was averaged as one biological replicate. This value from each of five embryos was used to calculate the overall mean across all the samples. The statistical significance of variation in the volume of bacteriocytes and nuclei was evaluated using ANOVA at a 0.05 level in Microsoft Excel.

### Data and Software Availability

The accession numbers for the raw reads of bacteriocyte genomes and head genomes reported in this paper are SRA: SRR6148276 (B1), SRA: SRR6148277 (B2), SRA: SRR6148278 (H1), and SRA: SRR6148279 (H2) (https://www.ncbi.nlm.nih.gov/sra). The accession numbers for the raw reads of bacteriocyte transcriptomes in nymph whiteflies [4] and adult whiteflies [13] reported in this paper are SRA: SRR1523521, SRA: SRR2001505. Other data supporting this study are provided within the paper and Supplemental Information.

## Supplemental Information

Document S1. Figure S1 and Tables S1–S4

Data S1. Microsatellite Profiles of Bacteriocytes and Heads of Whiteflies, Related to Figure 1 S1A. From different adult female whiteflies for ten microsatellite markers. S1B. From the same adult female whiteflies for five microsatellite markers, B, bacteriocyte; H, head. The minor peaks ahead of the main peak indicate contamination by other body of insects during dissection or stutter, so they are omitted.

Data S2. Microsatellite Profiles in Ten Cross Experiments (A–J) over Three Generations (F0, F1, and F2) for Five Microsatellite Markers, Related to Figure 2 B: bacteriocyte of female; H: head of female; M: head of male. The minor peaks ahead of the main peak are interpreted either as stutter or as contamination by other body tissues during dissection, and they are omitted.

Document S2. Article plus Supplemental Information
