# Supplementary material for: Maternal Inheritance of a Single Somatic Animal Cell Displayed by the Bacteriocyte in the Whitefly Bemisia tabaci
Source: Curr Biol. 2018 Feb 5;28(3):459–465.e3. doi: 10.1016/j.cub.2017.12.041 (PMC5807091; doi:10.1016/j.cub.2017.12.041)
Supplement: Document S1. Figure S1 and Tables S1–S4 [file mmc1.pdf]

Current Biology, Volume *28*

Supplemental Information

Maternal Inheritance of a Single Somatic Animal Cell Displayed by the Bacteriocyte in the Whitefly *Bemisia tabaci*

Junbo Luan, Xuepeng Sun, Zhangjun Fei, and Angela E. Douglas

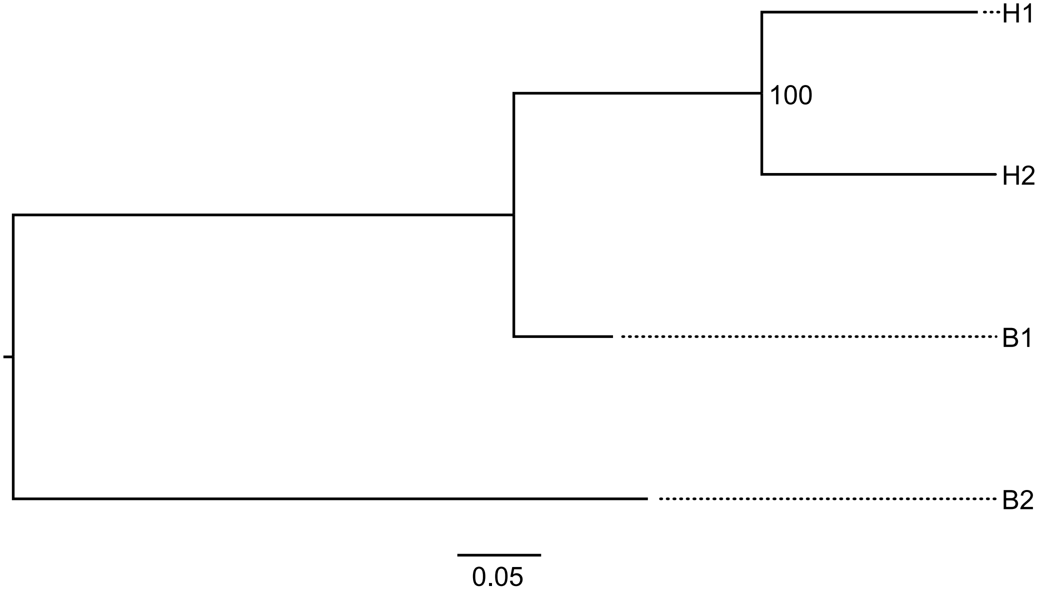

Figure S1. Maximum likelihood phylogeny of bacteriocytes (B1 and B2) and heads (H1 and H2) using all biallelic SNPs (Related to Figure 3 and Table S3). B1 and H1 were isolated from an individual female whitefly, and B2 and H2 from another female whitefly. The two whiteflies were haphazardly collected in the population. The tree is mid-point rooted, and the number on branch is the bootstrap value.

Microsatellite markers	Microsatellite	Primer sequence (5'–3')	Repeat motif	Fluorescent dye color (Applied Biosystems)	Accession numbers	Allele size range (bp) in our lab population	Location in whitefly genome (with the highest identity)
**Population genotyping**	WF1B11	F: GCATTGAACATTTTTCTGCATGCGCG	$(CCTGA)_{12}$ imp	FAM	JQ436838	136-168	Scaffold54198
		R: GCACACAGCTCTCCAAAAGAAAGGTC					
	WF2H06	F: TATTCGCCAATCGATTCCTT	$(TTTG)_{11}$ imp	FAM	JQ436844	172–196	Scaffold684
		R: CGGCGGAAATTTCGATAAA					
	WF2A02	F: GAAGTGCATAAAAACATCGTCGCCG	$(CTTT)_{10}$ imp	PET	JX853746	155	Scaffold1034
		R: CAATTGAAAGGCTAGACTCCCTGGC					
	WF1D04	F: GTTGTTAGGTTACAGGGTTTGTC	$(CAAA)_{16}$	PET	JQ436839	124-168	Scaffold4012
		R: GTCTTTACTTCTCTTTTCCTCCG					
	WF1G03	F: CTCCAAAATGGGACTTGAAC	$(GTTT)_{8}$	NED	JQ436840	158	Scaffold147
		R: GTAGAAGCCACACATACTAGCAC					
	WF2C01	F: ATGATACCGCACGAAAAAGAGGACG	$(GTTT)_{11}$ imp	NED	JQ436842	150-188	Scaffold29488
		R: CTTGAATTACATCAAACGCAGCAGC					
	WF2A05	F: ATTGATCATTTTCGGCTACCTTATC	$(CAAA)_{12}$ imp	VIC	JQ436841	147-155	Scaffold519
		R: CGACCCTTCTGTAAATGATAGACT					
	BtIs1.11	F: ATGTTATGACTATCGCAATC	$(CA)_{8}$	VIC	BV726565	119-133	Scaffold3874
		R: GCAGTGTGTAAGGGTGTC					
	BtIs1.1	F: CCCATAGAACACGCTCC	$(CA)_{7}$	VIC	BV726563	231–261	Scaffold651
		R: CATTGGAAGCCTCGAATAC					
	WF2E11	F: TCTCCAACCATAATTTTTAATCTCG	$(GATT)_{27}$ imp	VIC	JQ436843	244-264	Scaffold130
		R: GTCTGGGCAGGAAAACGAT					
**Cross experiments**	WF2C01	F: ATGATACCGCACGAAAAAGAGGACG	$(GTTT)_{11}$ imp	FAM	JQ436842	150-188	Scaffold29488
		R: CTTGAATTACATCAAACGCAGCAGC					
	WF2H06	F: TATTCGCCAATCGATTCCTT	$(TTTG)_{11}$ imp	PET	JQ436844	172–196	Scaffold684
		R: CGGCGGAAATTTCGATAAA					
	WF1B11	F: GCATTGAACATTTTTCTGCATGCGCG	$(CCTGA)_{12}$ imp	NED	JQ436838	136-168	Scaffold54198
		R: GCACACAGCTCTCCAAAAGAAAGGTC					
	WF1D04	F: GTTGTTAGGTTACAGGGTTTGTC	$(CAAA)_{16}$ imp	VIC	JQ436839	124-168	Scaffold4012
		R: GTCTTTACTTCTCTTTTCCTCCG					
	WF2E11	F: TCTCCAACCATAATTTTTAATCTCG	$(GATT)_{27}$ imp	VIC	JQ436843	244-264	Scaffold130
		R: GTCTGGGCAGGAAAACGAT					

Table S1. Microsatellite loci and microsatellite primers (Related to Figure 1,2 and Data S1,2 and Table S2).

Cross ID	Generations	Markers	Sequences
C	F0	WF2E11	GATTGATTGATTGATTGATTGATTGATTGATTGATTGATTGATTGATTGATTGATTGAATGATTGATTGATTGATTGAATGATTGATTTATTGATTGATTGATTGATTTATTGATTGATGGATCACATCAAGTGAAAATGTGTTTCATTTACCGAAAAAGTGATG
C	F1	WF2E11	GATTGATTGATTGATTGATTGATTGATTGATTGATTGATTGATTGATTGATTGATTGAATGATTGATTGATTGATTGAATGATTGATTTATTGATTGATTGATTGATTTATTGATTGATGGATCACATCAAGTGAAAATGTGTTTCATTTACCGAAAAAGTGATG
C	F2	WF2E11	GATTGATTGATTGATTGATTGATTGATTGATTGATTGATTGATTGATTGATTGATTGAATGATTGATTGATTGATTGAATGATTGATTTATTGATTGATTGATTGATTTATTGATTGATGGATCACATCAAGTGAAAATGTGTTTCATTTACCGAAAAAGTGATG
F	F0	WF2E11	GATTGATTGATTGATTGATTGATTGATTGATTGATTGATTGATTGATTGATTGATTGAATGATTGATTGATTGATTGAATGATTGATTTATTGATTGATTGATTGATTTATTGATTGATGGATCACATCAAGTGAAAATGTGTTTCATTTACCGAAAAAGNGATG
F	F1	WF2E11	GATTGATTGATTGATTGATTGATTGATTGATTGATTGATTGATTGATTGATTGATTGAATGATTGATTGATTGATTGAATGATTGATTTATTGATTGATTGATTGATTTATTGATTGATGGATCACATCAAGTGAAAATGTGTTTCATTTACCGAAAAAGCGATG
F	F2	WF2E11	GATTGATTGATTGATTGATTGATTGATTGATTGATTGATTGATTGATTGATTGATTGAATGATTGATTGATTGATTGAATGATTGATTTATTGATTGATTGATTGATTTATTGATTGATGGATCACATCAAGTGAAAATGTGTTTCATTTTACCGAAAAAGTGATG
C	F0	WF1B11	CTTGACCTGACCTGACCTGACCTGACCTGATCTGACCTGACCTGACCTGACCTGACCTGACCTTT
C	F1	WF1B11	CTTGACCTGACCTGACCTGACCTGACCTGATCTGACCTGACCTGACCTGACCTGACCTGACCTTT
C	F2	WF1B11	CTTGACCTGACCTGACCTGACCTGACCTGATCTGACCTGACCTGACCTGACCTGACCTGACCTTT
F	F0	WF1B11	CTTGACCTGACCTGACCTGACCTGACCTGATCTGACCTGACCTGACCTGACCTGANCTGACCTTT
F	F1	WF1B11	CTTGACCTGACCTGGACCTGACCTGACCTGATCTGACCTGACCTGACCTGACCTGACCTGACCTTT
F	F2	WF1B11	CTTGACCTGACCTGACCTGACCTGACCTGATCTGACCTGACCTGACCTGACCTGACCTGACCTTT
C	F0	WF2H06	TTTTATCAGTCTCATTTTATCAAAACGCTCACCGGCTATTTGTTTGTTTGTTTGNTTGTTTGTTTGTTTGTTTGTTTGT
C	F1	WF2H06	TTTTATCAGTCTCATTTTATCAAAACGCTCACCGCTATTTGTTTGTTTGTTTGTTTGTTTGTTTGTTTGTTTGTTTGT
C	F2	WF2H06	TTTTATCAGTCTCATTTTATCAAAACGCTCACCGCTATTTGTTTGTTTGTTTGTTTGTTTGTTTGTTTGTTTGTTTGT
F	F0	WF2H06	TTTTATCAGTCTCATTTTATCAAAACGCTCACCGCTATTTGTTTGTTTGTTTGTTTGTTTGTTTGTTTGTTTGTTTGT
F	F1	WF2H06	TTTTATCAGTCTCATTTTATCAAAACGCTCACCGCTATTTGTTTGTTTGTTTGTTTGTTTGTTTGTTTGTTTGTTTGT
F	F2	WF2H06	TTTTATCAGTCTCATTTTATCAAAACGCTCACCGCTATTTGTTTGTTTGTTTGTTTGTTTGTTTGTTTGTTTGTTTGT
C	F0	WF2C01	GTTTGTTTGTTTGTTTGTTTGTTTGTTTGTTGGTTTGTTTGTTTGTTTGTTT
C	F1	WF2C01	GTTTGTTTGTTTGTTCGTTNGTTTGTTTGTTTGTTTGTTTGTTTGTTTGTTT
C	F2	WF2C01	GGTTGTTTGTTTGTTTGTTTGTTTGTTTGTTTGTTTGTTTGTTTGTTTGTTT
F	F0	WF2C01	GTTTGTTTGTTTGTTTGTTTGTTTGTTTGTTTGTTTGTTTGTTTGTTTGTTT
F	F1	WF2C01	GTTTGTTTGTTTGTTTGTTTGTTTGTTTGTTTGTTTGTTTGTTTGTTTGTTT
F	F2	WF2C01	GGTTGTTTGTTTGTTTGTTTGTTTGTTTGTTTGTTTGTTTGTTTGTTTGTTT
C	F0	WF1D04	NAAACAAACAAACAAACAAACAAACAAACAAACAAACAAACAAACAAA
C	F1	WF1D04	CAAACAAACAAACAAACAAACAAACAAACAAACAAACAAACAAACAAA
C	F2	WF1D04	CAAACAAACAAACAAACAAACAAACAAACAAACAAACAAACAAACAAA
F	F0	WF1D04	CAAACAAACAAACAAACAAACAAACAAACAAACAAACAAACAAACAAA
F	F1	WF1D04	CAAACAAACAAACAAACAAACAAACAAACAAACAAACAAACAAACAAA
F	F2	WF1D04	CAAACAAACAAACAAACAAACAAACAAACAAACAAACAAACAAACAAA

Table S2. Sequences of microsatellite loci derived by Sanger sequencing of PCR products of bacteriocytes isolated from adult female whiteflies in cross experiments C and F over three generations (Related to Figure 2 and Data S2).

	H1	H2	B1	B2
Raw reads				
#Read pairs	204,888,779	92,341,548	92,242,175	68,994,911
Total nucleotides (bp)	31,143,094,408	14,035,915,296	14,020,810,600	10,487,226,472
Cleaned & non-duplicated reads				
#Read pairs	55,144,510	38,543,693	23,046,421	48,724,623
Total nucleotides (bp)	8,265,564,207	5,784,811,536	3,459,552,051	7,322,190,441
Mean coverage	12.0×	8.4×	5.0×	10.6×
#SNPs	453,208			
#Biallelic	451,709			
#Homozygous	134,380	165,575	162,822	296,728
#Heterozygous	317,329	286,134	288,887	154,981
#Multiallelic	1,499			
#INDELs	60,348			

Table S3. Summary of genome resequencing and SNPs (Related to Figure 3).

Bacteriocyte B1 and head H1 are from one individual whitefly, and bacteriocyte B2 and head H2 from another individual whitefly.

Genes	Whitefly genome ID	NB_FPKM	AB_FPKM
Telomerase reverse transcriptase (TERT1)	Bta11058	1.00	1.41
Telomerase reverse transcriptase (TERT2)	Bta11059	2.37	2.62
Telomerase Cajal body protein 1 (TCAB1)	Bta15778	4.37	5.54
Telomerase-binding protein EST1A	Bta15441	15.34	15.75
Telomere length regulation protein TEL2	Bta09854	12.98	19.41

Table S4. Expression of telomere maintenance genes in whitefly bacteriocytes of both nymphs and adults (Related to Figure 3).

NB denotes bacteriocytes of nymph whiteflies and AB denotes bacteriocytes of adult whiteflies.

The data were obtained by mapping raw reads of bacteriocyte transcriptome [S1, S2] to whitefly genome [S3] and the FPKM value (fragments per kilobase of transcript per million fragments mapped) was calculated.

Supplemental References:

S1 Luan, J.B., Shan, H.W., Isermann, P., Huang, J.H., Lammerding, J., Liu, S.S., and Douglas, A.E. (2016). Cellular and molecular remodelling of a host cell for vertical transmission of bacterial symbionts. Proc. R. Soc. Lond. B. Biol. Sci. *283*, 20160580.

S2 Luan, J.B., Chen, W., Hasegawa, D.K., Simmons, A.M., Wintermantel, W.M., Ling, K.S., Fei, Z., Liu, S.S., and Douglas, A.E. (2015). Metabolic coevolution in the bacterial symbiosis of whiteflies and related plant sap-feeding insects. Genome Biol. Evol. *7*, 2635-2647.

S3 Chen, W., Hasegawa, D.K., Kaur, N., Kliot, A., Pinheiro, P.V., Luan, J., Stensmyr, M.C., Zheng, Y., Liu, W., Sun, H., et al. (2016). The draft genome of whitefly *Bemisia tabaci* MEAM1, a global crop pest, provides novel insights into virus transmission, host adaptation, and insecticide resistance. BMC Biol. *14*, 110.
