# Supplementary material for: Maternal Inheritance of a Single Somatic Animal Cell Displayed by the Bacteriocyte in the Whitefly Bemisia tabaci
Source: Curr Biol. 2018 Feb 5;28(3):459–465.e3. doi: 10.1016/j.cub.2017.12.041 (PMC5807091; doi:10.1016/j.cub.2017.12.041)
Supplement: Document S2. Article plus Supplemental Information [file mmc4.pdf]

Report

Current Biology

Maternal Inheritance of a Single Somatic Animal Cell Displayed by the Bacteriocyte in the Whitefly *Bemisia tabaci*

Graphical Abstract

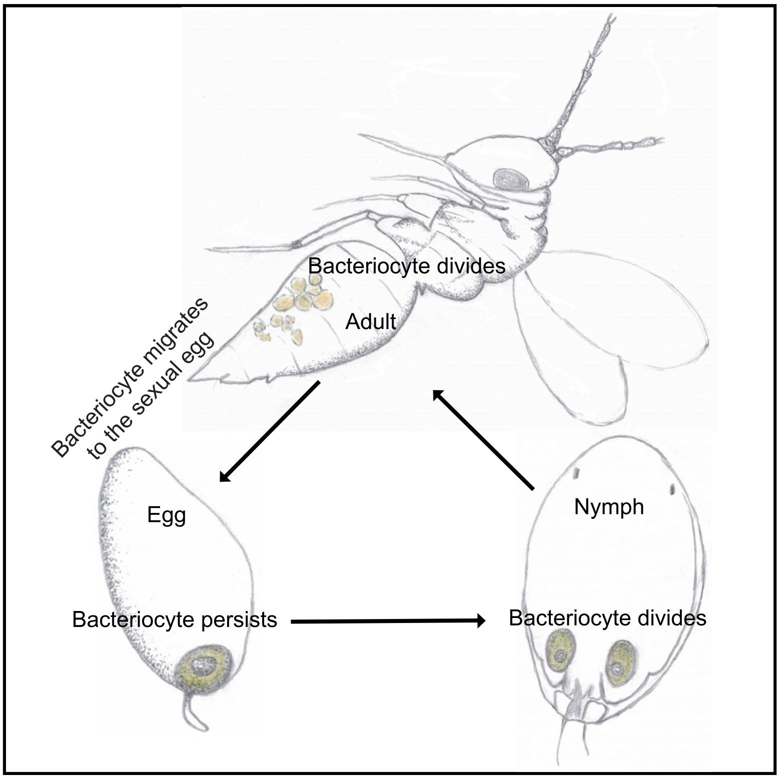

Highlights

- Whitefly bacteriocytes have different microsatellite alleles from other somatic cells
- The bacteriocyte genotype is stable over three sexual generations of the insect
- Bacteriocyte genomes in different insects are genetically very similar
- The single bacteriocyte transmitted to the sexual egg persists through embryogenesis

Authors

Junbo Luan, Xuepeng Sun, Zhangjun Fei, Angela E. Douglas

Correspondence

aes326@cornell.edu

In Brief

Luan et al. find that a cell bearing symbiotic bacteria is transferred to each sexual egg of whiteflies and retained through embryo development, leading to the maternal inheritance of a somatic cell with divergent genotype from other cells in the insect body. This mode of inheritance violates the germline/soma separation in animal development.

Luan et al., 2018, Current Biology *28*, 459–465
February 5, 2018 © 2017 The Author(s). Published by Elsevier Ltd.
https://doi.org/10.1016/j.cub.2017.12.041

Current Biology
Report

Maternal Inheritance of a Single Somatic Animal Cell Displayed by the Bacteriocyte in the Whitefly *Bemisia tabaci*

Junbo Luan,[1,5,6] Xuepeng Sun,[2,5] Zhangjun Fei,[2,3] and Angela E. Douglas[1,4,7,*]
[1]Department of Entomology, Cornell University, Ithaca, NY 14853, USA
[2]Boyce Thompson Institute, Ithaca, NY 14853, USA
[3]USDA-Agricultural Research Service, Robert W. Holley Center for Agriculture and Health, Ithaca, NY 14853, USA
[4]Department of Molecular Biology and Genetics, Cornell University, Ithaca, NY 14853, USA
[5]These authors contributed equally
[6]Present address: College of Plant Protection, Shenyang Agricultural University, Shenyang 110866, China
[7]Lead Contact
*Correspondence: aes326@cornell.edu
https://doi.org/10.1016/j.cub.2017.12.041

SUMMARY

Bacteriocytes are insect cells harboring symbiotic bacteria that are required by their insect host and are transmitted vertically via the female ovary [1]. In most insect groups, the bacteria are released from the bacteriocytes and transferred to the ovary [2, 3], but in whiteflies, maternal bacteriocytes migrate to each egg [4–6], where they have been reported to lyse, releasing the symbionts [1]. To investigate bacteriocyte inheritance in whiteflies further, we applied microsatellite genotyping and genomic analysis to a genetically diverse population of *Bemisia tabaci*, and we observed the fate of the bacteriocyte in embryos. Surprisingly, the microsatellite profile of the bacteriocytes was uniform, and insect cross experiments demonstrated that the bacteriocytes have a stable genotype that differs from the genotype of the insect head (which lacks bacteriocytes). Comparative genomic analysis indicates that genomes of the bacteriocyte and whitefly head are distinct. Interestingly, the bacterioyte genome contains the canonical arthropod telomere repeats TTAGG, and the bacteriocytes express telomere maintenance genes that may underlie cellular immortality in animal cells [7]. Microscopy observations confirmed that a single bacteriocyte transmitted to each egg is retained and divides once just before egg hatch, yielding two bacteriocytes in the neonate insect. These data demonstrate the maternal inheritance of an absolutely required somatic insect cell, violating the developmental separation of germline and soma [8, 9]. Future investigation on the mechanism and phylogenetic distribution of maternally inherited bacteriocytes will shed light on the developmental origins and evolutionary diversification of bacteriocytes [10] and the processes underlying cellular immortality [11].

RESULTS

Bacteriocytes Have the Same Microsatellite Alleles in the Whitefly Population

Our first experiment scored the genetic variation of whiteflies using 10 microsatellite markers (Table S1). Analysis of the heads (which have no bacteriocytes) of 10 female insects revealed polymorphisms in 6 of the 10 microsatellite loci (Figure 1A; Data S1A). In contrast, bacteriocytes dissected from a different haphazardly selected set of 10 female insects had a uniform microsatellite profile. To investigate the basis for this difference, we then determined the microsatellite profile from the head and bacteriocytes taken from the same insect. Across 7 replicate adult female insects, each of the five microsatellites tested yielded multiple genotypes for the heads but a single genotype for each microsatellite that was identical to that in the first analysis for the bacteriocytes (Figure 1B; Data S1B). Furthermore, one allele present in bacteriocytes was absent from the head samples and vice versa (Figure 1B: a, b, and e–g).

These data suggest that bacteriocytes in the whitefly population are genetically both more uniform than, and different from, other somatic tissues of the insects. Taken together with the microscopical evidence that a single whitefly bacteriocyte migrates from the body cavity to each unfertilized egg in the female ovary [1, 4], these data raise the possibility that the bacteriocytes may be maternally inherited.

Bacteriocytes Have Stable Genotypes over Three Sexual Generations of the Insect

To investigate whether bacteriocyte genotypes can be stably inherited over several generations, we designed 10 cross experiments using various combinations of whiteflies with diverse genotypes. Five polymorphic microsatellite loci were scored for female bacteriocytes, female heads, and male heads over three generations (Data S2). Because whiteflies are haplodiploid, we predicted that female offspring would have one maternal allele and one paternal allele, and this was observed for the head genotype in every female offspring tested in F1 and F2. In contrast, the bacteriocyte genotypes in the female offspring (F1 and F2) were identical to those in F0 female adult

This is an open access article under the CC BY license (http://creativecommons.org/licenses/by/4.0/).

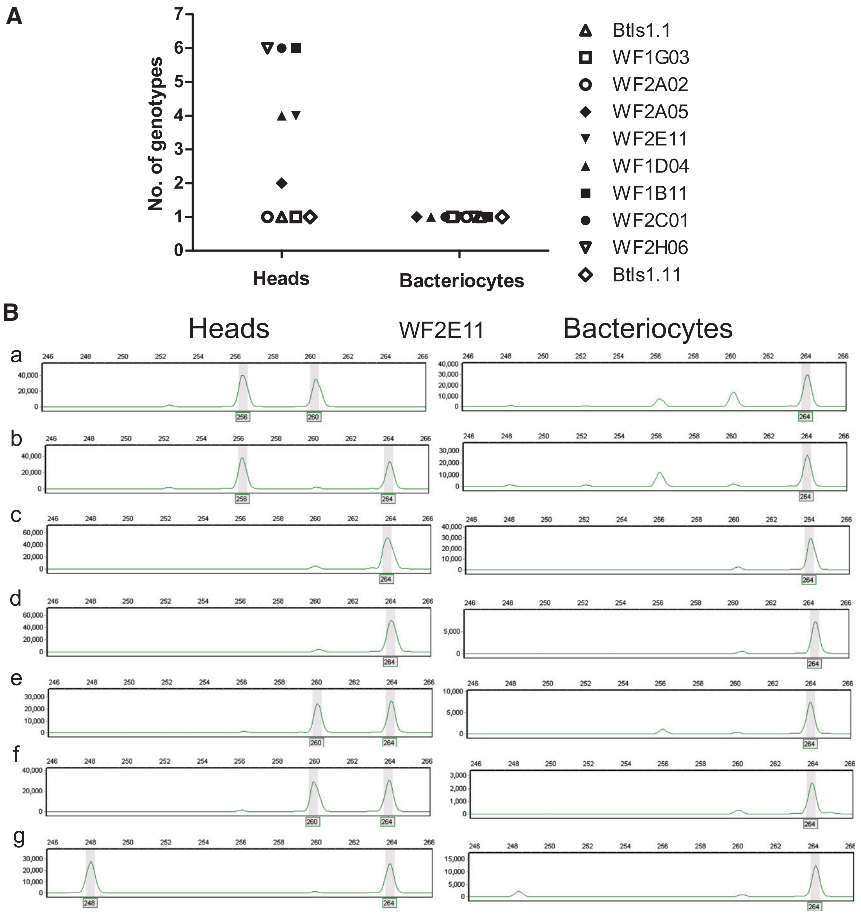

Figure 1. Genetic Variation of Bacteriocytes in the *Bemisia tabaci* Population

The insects were adult females haphazardly selected from the routine insect culture.

(A) Genetic variation of bacteriocytes and heads from different adult female whiteflies revealed by microsatellite profiles for ten markers. Two sets of ten insects were sampled, one for heads and the other for bacteriocytes.

(B) Genetic variation of bacteriocytes and heads from the same female adult whiteflies represented by microsatellite profiles for the marker WF2E11. Each head and bacteriocyte sample was collected from each of seven insects (a–g). The gray shading denotes the allele identified for this sample. The minor peaks with the sizes ranging from 248 to 260 in bacteriocytes are likely contamination by DNA from other insect tissues, which cannot be excluded completely during bacteriocyte dissections of these very small insects.

See also Table S1 and Data S1.

whiteflies (Figures 2A and 2B; Data S2). In some cases, the bacteriocyte and head alleles in a single insect were different (F1 in Figures 2A and 2B; Data S2). Furthermore, four alleles were detected in the bacteriocyte (but not head) samples with the microsatellite marker WF2H06 (Figure 2A), raising the possibility that the bacteriocyte nucleus may be polyploid.

To test for the possibility that the microsatellite primers amplified DNA from the maternally inherited bacterial symbionts, we conducted Sanger sequencing of PCR products for all five microsatellite loci in two of the cross experiments (cross-C and cross-F in Data S2) over three generations. All the products of microsatellite loci had 97%–100% of sequence identity to the whitefly genome and no detectable identity to the genome of either *Portiera* or *Hamiltonella*, the two endosymbiotic bacteria in the bacteriocytes (Table S2). These data demonstrate that the loci tested in the bacteriocyte samples are of insect origin.

We reasoned that, if the bacteriocytes (which reside in the insect abdomen) are the only cells that are somatically inherited, then the microsatellite profile of the insect abdomen should comprise both the alleles in the head and bacteriocyte. To test this prediction, we isolated the head, a subset of the bacteriocytes, and the abdomen with some remaining bacteriocytes from one female adult in the F2 offspring, and we tested the genotypes. In this individual, the head had two alleles and the bacteriocytes had one different allele for the microsatellite marker WF2E11. As expected, the abdomen including bacteriocytes contained all three alleles (Figure 2C).

Bacteriocyte Genome Is Distinct from Whitefly Genome

The observation above indicates that the bacteriocyte genome could be different from the genome of other somatic cells in whiteflies. To investigate this possibility, genome resequencing was conducted on the bacteriocytes and head dissected from each of two adult female whiteflies selected at random from the population. We generated 10.5–31.1 Gb raw data for the four samples (two bacteriocyte samples and two head samples). After removing low-quality and adaptor sequences and collapsing duplicated reads, we obtained 3.5–8.3 Gb final cleaned sequences (mean coverage 5–12×; Table S3), which were used for variant calling with the *B. tabaci* MEAM1 genome as a reference [12]. A total of 513,556 variants were identified among these four samples, which included 453,208 SNPs (biallelic, 451,709; multiallelic, 1,499) and 60,348 small insertions or deletions (indels) (Table S3).

To infer the genetic distance between the samples, we constructed a phylogenetic tree using all biallelic SNPs. The tree showed that the two head samples clustered together and separated from bacteriocyte samples (Figure S1). We reasoned that some of the heterozygous calls in bacteriocytes could be due to contamination of the dissected bacteriocytes by other tissues (see legend of Figure 1). We therefore reconstructed the phylogeny using variants comprising biallelic SNPs that are homozygous in every sample (1,775 sites). This tree indicated that the two bacteriocytes were closely related, and the two head samples were relatively more distant from each other than the bacteriocytes (Figure 3A). This observation is consistent with results from microsatellite analyses (Figures 1 and 2). In conclusion, the genomic evidence further confirms that

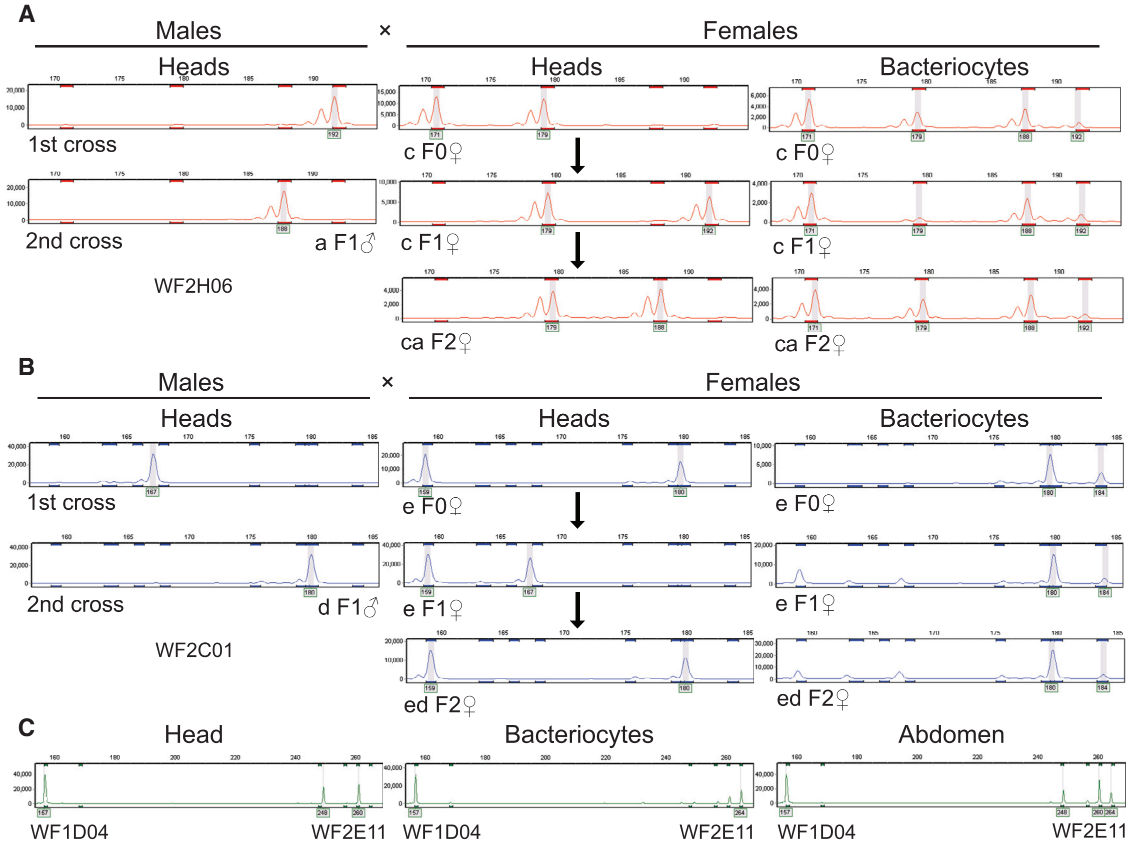

Figure 2. Genetic Variation of Bacteriocytes over Three Sexual Generations of *Bemisia tabaci*

(A and B) Representative microsatellite profiles in male heads, female heads, and female bacteriocytes of adult whiteflies for the microsatellite marker WF2H06 in cross C (A) and for the microsatellite marker WF2C01 in cross F (B) over three generations. The bacteriocytes and heads were isolated from the same adult female whiteflies (data for all microsatellite loci are provided in Data S2). The arrows refer to generation of female progeny by crossing one female and one male. The gray shading denotes the allele identified for this sample. The minor peaks with the sizes ranging from 159 to 167 in (B) are likely contamination by other insect tissues (see legend to Figure 1) and peak stutter.

(C) Microsatellite profiles for the markers WF1D04 and WF2E11 in the head, bacteriocytes, and abdomen of the same F2 adult whitefly in cross B. The gray shading denotes the allele identified for this sample. The minor peaks with the sizes ranging from 248 to 260 indicate possible contamination by other body tissue of insects during dissection and peak stutter.

See also Tables S1 and S2 and Data S2.

bacteriocytes are not inherited in parallel with other somatic cells, as represented by the insect heads.

Bacteriocytes Possess Canonical Arthropod Telomere Repeats of TTAGG and Express Telomere Maintenance Genes

In addition to being maternally inherited (as shown above), bacteriocytes are actively dividing cells, with an estimated 8–10 cell divisions over the lifespan of an individual insect [4]. We predicted that this remarkable cellular immortality is associated with the expression of telomerase, which stabilizes chromosomal telomeres in other immortal cells, including stem cells, germ cells, and many cancer cells; in normal soma cells, telomerase is not expressed, and these cells lose a portion of the telomere at each mitosis and eventually die [7, 14].

By analyzing the genome resequencing reads, we found that the bacteriocyte genome contained the canonical telomere repeat $(TTAGG)_n$, which is the ancestral condition for insects and present in members of the order Hemiptera (which includes whiteflies) investigated to date [15, 16]. We also quantified the expression of genes with key functions in telomere maintenance, including those encoding the following: telomerase reverse transcriptase (TERT), which mediates the addition of nucleotides in a TTAGG sequence to telomeres; telomerase Cajal body protein 1 (TCAB1), required for telomere trafficking and synthesis in cancer cells; telomerase-binding protein EST1A; and telomere length regulation protein TEL2, using bacteriocyte transcriptome sequencing data of both nymphs and adults [4, 13]. All these genes were expressed in the bacteriocytes of both nymphs and adults of whiteflies (Figure 3B; Table S4). Taken together, these data suggest that the immortality of the bacteriocytes in *B. tabaci* may be underlain by the retention of the $(TTAGG)_n$ telomere repeat and expression of telomere maintenance genes.

Dynamics of Bacteriocytes and Associated Nuclei during Whitefly Embryogenesis

To investigate the cellular basis of the maternal inheritance of whitefly bacteriocytes, we monitored the dynamics of

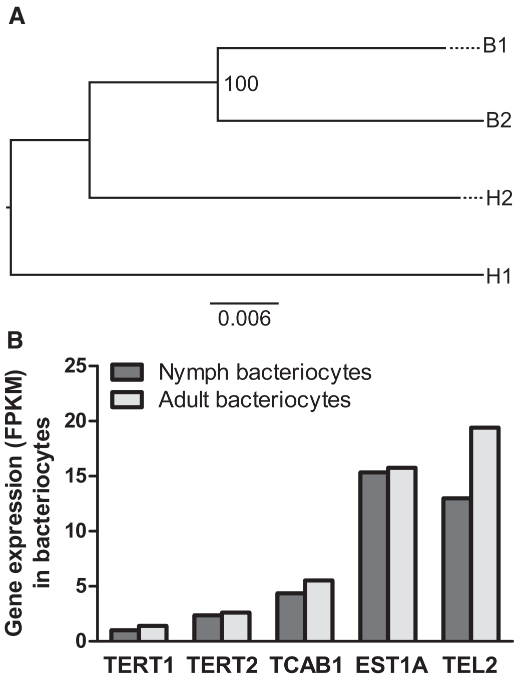

Figure 3. Bacteriocytes Have the Genome Distinct from Whitefly Genome Represented by Heads and Express Telomere Maintenance Genes

(A) Genetic distance of bacteriocytes (B1 and B2) and head (H1 and H2) from two individuals shown by maximum likelihood phylogeny. B1 and H1 are from one individual whitefly and B2 and H2 are from another individual whitefly. The tree was constructed using homozygous SNPs and is mid-point rooted. The number on branch is the bootstrap value.

(B) Expression of telomere maintenance genes in nymph bacteriocytes and adult bacteriocytes. The data were obtained by mapping raw reads of bacteriocyte transcriptome [4, 13] to the whitefly genome [12] and the FPKM value (fragments per kilobase of transcript per million fragments mapped) was calculated.

See also Figure S1 and Tables S3 and S4.

bacteriocytes during whitefly embryogenesis (Figure 4). A single bacteriocyte was localized to the posterior region of newly deposited eggs at day 0 (1 hr post-oviposition). This bacteriocyte subsequently took up a medial location at day 4, and it increased progressively in size at 5–6 days post-oviposition. By day 7, the bacteriocyte returned to the posterior pole, prior to cell division. All embryos just prior to hatch (day 8; Figure 4A) and neonate nymphs [4] bore two bacteriocytes. The onset of bacteriocyte division between day 7 and day 8 was coincident with an approximate halving of the bacteriocyte volume (Figure 4B) and a significant reduction in size of the nucleus (Figure 4C). The mean volume of other nuclei in the embryo was both much smaller than that of bacteriocyte nuclei and did not vary significantly with developmental age (Figure 4C). Taken together, our microscopy observations confirm the genetic and molecular evidence that maternal bacteriocytes are inherited by the next insect generation.

DISCUSSION

According to conventional wisdom, the separation of the germline and soma is crucial for sustained cooperation among cells of animals and other multicellular organisms [9, 17, 18]. It is argued that, because all somatic cells are derived from the zygote nucleus, they are genetically identical and, therefore, have common genetic interests. The importance of the soma/germline separation is illustrated by the principal known exceptions: various cancer cells, whose immortality is deleterious to the individual. Although most cancers die with their host, there are rare instances of maternal inheritance of leukemia cells in humans [19], as well as lineages of contagious tumor cells in dogs and the Tasmanian devil *Sarcophilus harrisii* [20, 21].

Despite the predicted evolutionary conflicts, we provide multiple lines of evidence to support that the bacteriocytes in the whitefly *B. tabaci* are somatically inherited: the incongruent microsatellite alleles between bacteriocytes and somatic tissues (the insect head, which is bacteriocyte free), the strict inheritance of bacteriocyte alleles through sexual generations of the insect, the genomic differences between the bacteriocytes and head of individual insects, and the continuity of the bacteriocyte through embryo development. The fate of bacteriocytes in embryos of *B. tabaci* has not been investigated previously, and our results differ from an early microscopical study of symbiont transmission in another whitefly species, *Aleurodes proletella*, where multiple bacteriocytes transferred to each egg are reported to degrade in the embryo, followed by incorporation of the symbiotic bacteria into recently differentiated bacteriocytes of embryo origin [1]. Although the analysis of *A. proletella* was not supported by any genetic data, this likely difference points to two different fates of maternal bacteriocytes in embryos of different whitefly species. A parsimonious interpretation is that the somatic inheritance of bacteriocytes in *B. tabaci* may have evolved from the condition reported for *A. proletella*. This evolutionary transition may not be unique, and future studies focusing on the Coccoidea (hemipteran insects related to whiteflies) may be particularly fruitful [22]. In particular, an early study is suggestive of the somatic inheritance of bacteriocytes in the coccid *Puto* [1]. A different developmental arrangement occurs in the coccid *Pseudococcus* and diaspidid coccids, where the bacteriocyte is pentaploid, being derived anew in each insect generation from the fusion of the three polar bodies generated during oocyte development with the diploid nucleus of one embryo cell [23–25].

Further research is required to establish the antiquity of the bacteriocyte lineage in *B. tabaci*. As for asexual lineages in general, the bacteriocyte lineage may be evolutionarily short-lived as a result of accumulating deleterious mutations [26, 27]. Countering this process, however, is the very strong selection for bacteriocyte function. Whiteflies feed on plant phloem sap, which is grossly deficient in essential amino acids [28], and insect fitness is absolutely dependent on the sustained overproduction of essential amino acids by the symbiotic bacterium *Portiera* maintained within the bacteriocytes [13]. Because a single bacteriocyte is transmitted to each whitefly offspring, each individual bacteriocyte is exposed to selection at each insect generation. In this way, bacteriocytes with deleterious mutations are eliminated, and, potentially, mutations that specifically enhance symbiosis function (but are selectively neutral or deleterious in other somatic cells) may be favored. Candidates for positive selection in bacteriocytes are metabolism genes that are expressed out of their normal metabolic context to contribute to shared metabolic

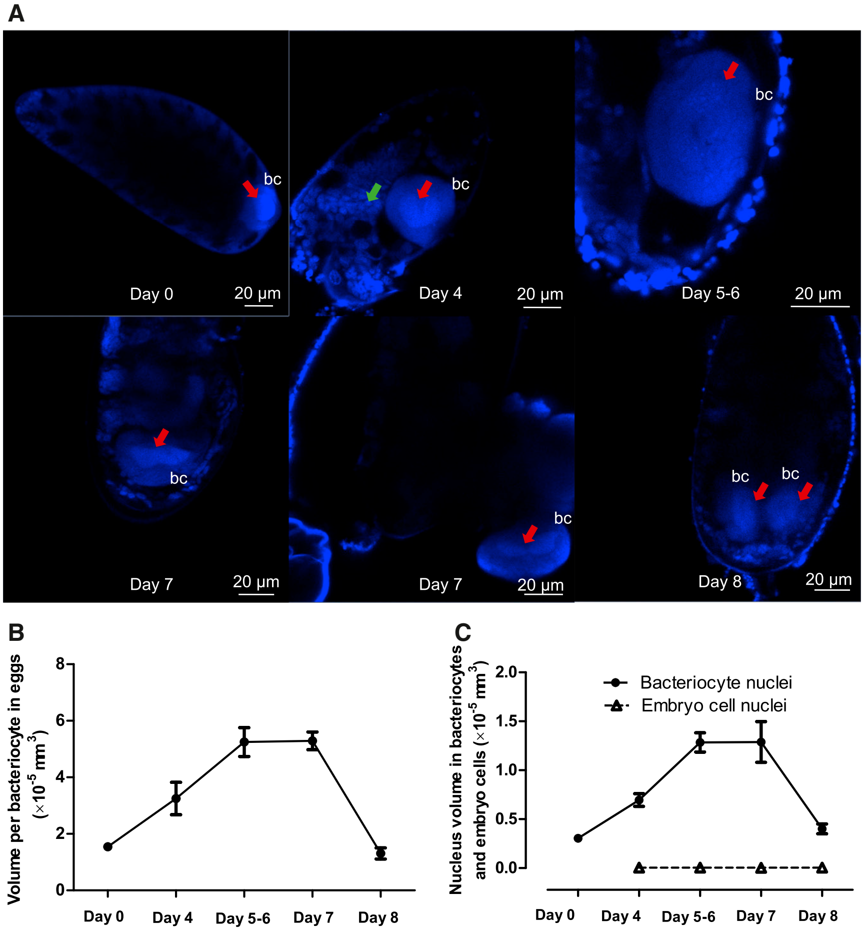

Figure 4. Dynamics of Bacteriocytes and Associated Nuclei during Whitefly Embryogenesis

(A) Localization of bacteriocytes and associated nuclei in eggs at 0, 4, 5–6, 7, and 8 days post-oviposition, revealed by Hoechst 33342 staining of DNA. The bacterial symbionts that pack the cytoplasm of bacteriocytes are evident in the bacteriocyte periphery. bc, bacteriocyte; red arrow, bacteriocyte nucleus; green arrow, egg nucleus.

(B) Bacteriocyte volume in eggs at 0, 4, 5–6, 7, and 8 days post-oviposition. Bacteriocyte volume was calculated from the diameter, assuming that the cell is a sphere, and it varied significantly with time (ANOVA: $F_{4,24}$ = 25.06, p < 0.001). Data are represented as mean ± SEM (5 replicates).

(C) Nucleus volume of the bacteriocytes (solid line) and embryo cells (broken line) in eggs at 0, 4, 5–6, 7, and 8 days post-oviposition. The volume of the bacteriocyte nuclei varied significantly with time (ANOVA: $F_{4,24}$ = 18.57, p < 0.001), but the volume of nuclei of other cells in the embryos did not vary significantly (ANOVA: $F_{3,19}$ = 3.98, p = 0.027). Data are represented as mean ± SEM (5 replicates).

pathways with the bacterial symbiont [13]. Long-term persistence of the bacteriocyte lineage may also be promoted by genetic exchange (by mechanisms that are currently unknown), as occurs in some ancient animal lineages without canonical sex [29, 30].

Pertinent to the discussion of the antiquity of maternally inherited bacteriocytes in *B. tabaci* is the architecture of the bacteriocyte genome. The very large size of the bacteriocyte nucleus suggests that the bacteriocyte genome may be polyploid, likely through genome endoreduplication (i.e., multiple rounds of genome replication without cell division), as occurs commonly in other insect somatic cells, including bacteriocytes of other species [31–33]. Mutations in a persistently polyploid genome (possibly accompanied by genetic exchange, as proposed above) are predicted to lead to genetic diversification, but extensive genetic variation was not evident in our analysis of the microsatellite loci of the bacteriocytes. A priority for further research is to quantify the number of genome copies in each bacteriocyte nucleus and to estimate the scale of genetic variation among the genome copies. A related issue is the developmental origin of the bacteriocyte in *B. tabaci*. These cells may have evolved from the putative ancestral whitefly bacteriocyte (which develops *de novo* in each insect generation, as reported in *A. proletella*) by suppression of maternal bacteriocyte degradation in the embryo. Alternatively, the bacteriocytes in *B. tabaci* may have a distinctive developmental origin, possibly derived from a whitefly cell lineage that gained immortality through somatic mutation(s) and subsequently gained dramatically enhanced fitness by incorporating the obligate symbionts. Under either scenario, conflict between the bacteriocyte lineage and cells of sexual origin is suppressed by their mutual dependence on sustained bacterial function.

In summary, the maternal inheritance of the bacteriocyte in the whitefly *B. tabaci* represents a remarkable exception to the strict separation of the germline and soma, a central tenet of Weismann's "doctrine of the continuity of the germline" [9]. Further investigation into the molecular and selective processes by which the whitefly bacteriocyte persists will provide new insights into the genetic basis of the individual in animals and the (im)mortality of cell lineages [11, 34], as well as contributing to our understanding of coevolved mutualisms between animals and their bacterial symbionts.

STAR★METHODS

Detailed methods are provided in the online version of this paper and include the following:

- KEY RESOURCES TABLE
- CONTACT FOR REAGENT AND RESOURCE SHARING
- EXPERIMENTAL MODEL AND SUBJECT DETAILS
- METHOD DETAILS
  - Genetic variation of bacteriocytes in the whitefly population
  - Genetic variation of bacteriocytes over three generations in cross experiments
  - Library construction and genome resequencing

- ○ Variant calling
- ○ Expression of telomere maintenance genes in bacteriocytes
- ○ Observation of bacteriocytes and nuclei during whitefly embryogenesis
- ● QUANTIFICATION AND STATISTICAL ANALYSIS
  - ○ Analysis of volume of bacteriocytes and nuclei
- ● DATA AND SOFTWARE AVAILABILITY

SUPPLEMENTAL INFORMATION

Supplemental Information includes one figure, four tables, and two data files and can be found with this article online at https://doi.org/10.1016/j.cub.2017.12.041.

ACKNOWLEDGMENTS

We thank Seung Ho Chung (Department of Entomology, Cornell University) for assistance with the design of the microsatellite experiments and Steve Bogdanowicz (Department of Ecology and Evolutionary Biology, Cornell University) and Andres Jose (Department of Biology, University of Saskatchewan, Canada) for helpful discussions on the microsatellite analysis. This work was funded by the Natural Resources Institute, University of Greenwich from a grant provided by the Bill & Melinda Gates Foundation (grant agreement OPP1058938) and the USAID Feed-the-Future Program (58-0210-3-012).

AUTHOR CONTRIBUTIONS

J.L. and A.E.D. designed the research. J.L. conducted all the experimental work. J.L. analyzed microsatellite profile data and bacteriocyte dynamics. X.S. analyzed the genomic sequence data. X.S. and J.L. analyzed the expression of telomere maintenance genes. Z.F. supervised the bioinformatics analysis. J.L., A.E.D., and X.S. drafted the first manuscript. All authors contributed to amending the manuscript and read the submitted version.

DECLARATION OF INTERESTS

The authors declare no competing interests.

Received: October 17, 2017
Revised: November 18, 2017
Accepted: December 18, 2017
Published: January 25, 2018

REFERENCES

1. Buchner, P. (1965). Endosymbiosis of Animals with Plant Microorganisms (Chichester, UK: John Wiley & Sons).
2. Koga, R., Meng, X.Y., Tsuchida, T., and Fukatsu, T. (2012). Cellular mechanism for selective vertical transmission of an obligate insect symbiont at the bacteriocyte-embryo interface. Proc. Natl. Acad. Sci. USA *109*, E1230–E1237.
3. Douglas, A.E. (1989). Mycetocyte symbiosis in insects. Biol. Rev. Camb. Philos. Soc. *64*, 409–434.
4. Luan, J.B., Shan, H.W., Isermann, P., Huang, J.H., Lammerding, J., Liu, S.S., and Douglas, A.E. (2016). Cellular and molecular remodelling of a host cell for vertical transmission of bacterial symbionts. Proc. Biol. Sci. *283*, 20160580.
5. Szklarzewicz, T., and Moskal, A. (2001). Ultrastructure, distribution, and transmission of endosymbionts in the whitefly *Aleurochiton aceris* Modeer (Insecta, Hemiptera, Aleyrodinea). Protoplasma *218*, 45–53.
6. Coombs, M.T., Costa, H.S., De Barro, P., and Rossell, R.C. (2007). Pre-imaginal egg maturation and bacteriocyte inclusion in *Bemisia* aff. *gigantea* (Hemiptera: Aleyrodidae). Ann. Entomol. Soc. Am. *100*, 736–744.
7. Maciejowski, J., and de Lange, T. (2017). Telomeres in cancer: tumour suppression and genome instability. Nat. Rev. Mol. Cell Biol. *18*, 175–186.
8. Swartz, S.Z., and Wessel, G.M. (2015). Germ line versus soma in the transition from egg to embryo. Curr. Top. Dev. Biol. *113*, 149–190.
9. Weismann, A. (1892). Das Keimplasma. Eine Theorie der Vererbung (Jena, Germany: Fischer).
10. Braendle, C., Miura, T., Bickel, R., Shingleton, A.W., Kambhampati, S., and Stern, D.L. (2003). Developmental origin and evolution of bacteriocytes in the aphid-Buchnera symbiosis. PLoS Biol. *1*, E21.
11. Hayflick, L. (2000). The illusion of cell immortality. Br. J. Cancer *83*, 841–846.
12. Chen, W., Hasegawa, D.K., Kaur, N., Kliot, A., Pinheiro, P.V., Luan, J., Stensmyr, M.C., Zheng, Y., Liu, W., Sun, H., et al. (2016). The draft genome of whitefly *Bemisia tabaci* MEAM1, a global crop pest, provides novel insights into virus transmission, host adaptation, and insecticide resistance. BMC Biol. *14*, 110.
13. Luan, J.B., Chen, W., Hasegawa, D.K., Simmons, A.M., Wintermantel, W.M., Ling, K.S., Fei, Z., Liu, S.S., and Douglas, A.E. (2015). Metabolic coevolution in the bacterial symbiosis of whiteflies and related plant sap-feeding insects. Genome Biol. Evol. *7*, 2635–2647.
14. O'Sullivan, R.J., and Karlseder, J. (2010). Telomeres: protecting chromosomes against genome instability. Nat. Rev. Mol. Cell Biol. *11*, 171–181.
15. Gomes, N.M., Shay, J.W., and Wright, W.E. (2010). Telomere biology in Metazoa. FEBS Lett. *584*, 3741–3751.
16. Kuznetsova, V.G., Grozeva, S.M., Hartung, V., and Anokhin, B.A. (2015). First evidence for (TTAGG)n telomeric sequence and sex chromosome post-reduction in Coleorrhyncha (Insecta, Hemiptera). Comp. Cytogenet. *9*, 523–532.
17. Maynard Smith, J., and Szathmary, E. (1995). The Major Transitions in Evolution (Oxford, UK: Oxford University Press).
18. Michod, R.E. (1999). Darwinian Dynamics: Evolutionary Transitions in Fitness and Individuality (Princeton, NJ: Princeton University Press).
19. Isoda, T., Ford, A.M., Tomizawa, D., van Delft, F.W., De Castro, D.G., Mitsuiki, N., Score, J., Taki, T., Morio, T., Takagi, M., et al. (2009). Immunologically silent cancer clone transmission from mother to offspring. Proc. Natl. Acad. Sci. USA *106*, 17882–17885.
20. Murgia, C., Pritchard, J.K., Kim, S.Y., Fassati, A., and Weiss, R.A. (2006). Clonal origin and evolution of a transmissible cancer. Cell *126*, 477–487.
21. Murchison, E.P., Schulz-Trieglaff, O.B., Ning, Z., Alexandrov, L.B., Bauer, M.J., Fu, B., Hims, M., Ding, Z., Ivakhno, S., Stewart, C., et al. (2012). Genome sequencing and analysis of the Tasmanian devil and its transmissible cancer. Cell *148*, 780–791.
22. Normark, B.B. (2003). The evolution of alternative genetic systems in insects. Annu. Rev. Entomol. *48*, 397–423.
23. Schrader, F. (1923). The origin of the mycetocytes in Pseudococcus. Biol. Bull. *45*, 279–302.
24. Nur, U. (1977). Maternal inheritance of enzymes in the mealybug *Pseudococcus obscurus* (Homoptera). Genetics *86*, 149–160.
25. Normark, B.B. (2004). The strange case of the armored scale insect and its bacteriome. PLoS Biol. *2*, E43.
26. Felsenstein, J. (1974). The evolutionary advantage of recombination. Genetics *78*, 737–756.
27. Muller, H.J. (1932). Some genetic aspects of sex. Am. Nat. *66*, 118–138.
28. Akman Gündüz, E., and Douglas, A.E. (2009). Symbiotic bacteria enable insect to use a nutritionally inadequate diet. Proc. Biol. Sci. *276*, 987–991.
29. Debortoli, N., Li, X., Eyres, I., Fontaneto, D., Hespeels, B., Tang, C.Q., Flot, J.F., and Van Doninck, K. (2016). Genetic exchange among bdelloid rotifers is more likely due to horizontal gene transfer than to meiotic sex. Curr. Biol. *26*, 723–732.
30. Signorovitch, A., Hur, J., Gladyshev, E., and Meselson, M. (2015). Allele sharing and evidence for sexuality in a mitochondrial clade of bdelloid rotifers. Genetics *200*, 581–590.

31. Edgar, B.A., Zielke, N., and Gutierrez, C. (2014). Endocycles: a recurrent evolutionary innovation for post-mitotic cell growth. Nat. Rev. Mol. Cell Biol. *15*, 197–210.

32. Lee, H.O., Davidson, J.M., and Duronio, R.J. (2009). Endoreplication: polyploidy with purpose. Genes Dev. *23*, 2461–2477.

33. Orr-Weaver, T.L. (2015). When bigger is better: the role of polyploidy in organogenesis. Trends Genet. *31*, 307–315.

34. Buss, L.W. (1983). Evolution, development, and the units of selection. Proc. Natl. Acad. Sci. USA *80*, 1387–1391.

35. Bolger, A.M., Lohse, M., and Usadel, B. (2014). Trimmomatic: a flexible trimmer for Illumina sequence data. Bioinformatics *30*, 2114–2120.

36. Li, H., and Durbin, R. (2009). Fast and accurate short read alignment with Burrows-Wheeler transform. Bioinformatics *25*, 1754–1760.

37. Li, H., Handsaker, B., Wysoker, A., Fennell, T., Ruan, J., Homer, N., Marth, G., Abecasis, G., and Durbin, R.; 1000 Genome Project Data Processing Subgroup (2009). The sequence alignment/map format and SAMtools. Bioinformatics *25*, 2078–2079.

38. Garrison, E., and Marth, G. (2012). Haplotype-based variant detection from short-read sequencing. arXiv, arXiv:1207.3907, https://arxiv.org/abs/1207.3907.

39. McKenna, A., Hanna, M., Banks, E., Sivachenko, A., Cibulskis, K., Kernytsky, A., Garimella, K., Altshuler, D., Gabriel, S., Daly, M., and DePristo, M.A. (2010). The Genome Analysis Toolkit: a MapReduce framework for analyzing next-generation DNA sequencing data. Genome Res. *20*, 1297–1303.

40. Nguyen, L.-T., Schmidt, H.A., von Haeseler, A., and Minh, B.Q. (2015). IQ-TREE: a fast and effective stochastic algorithm for estimating maximum-likelihood phylogenies. Mol. Biol. Evol. *32*, 268–274.

41. Zheng, X., Levine, D., Shen, J., Gogarten, S.M., Laurie, C., and Weir, B.S. (2012). A high-performance computing toolset for relatedness and principal component analysis of SNP data. Bioinformatics *28*, 3326–3328.

42. Dobin, A., Davis, C.A., Schlesinger, F., Drenkow, J., Zaleski, C., Jha, S., Batut, P., Chaisson, M., and Gingeras, T.R. (2013). STAR: ultrafast universal RNA-seq aligner. Bioinformatics *29*, 15–21.

43. Gauthier, N., Dalleau-Clouet, C., and Bouvret, M.E. (2008). Twelve new polymorphic microsatellite loci and PCR multiplexing in the whitefly, *Bemisia tabaci*. Mol. Ecol. Resour. *8*, 1004–1007.

44. Hadjistylli, M., Schwartz, S.A., Brown, J.K., and Roderick, G.K. (2014). Isolation and characterization of nine microsatellite loci from *Bemisia tabaci* (Hemiptera: Aleyrodidae) biotype B. J. Insect Sci. *14*, 148.

45. Luan, J.B., Ghanim, M., Liu, S.-S., and Czosnek, H. (2013). Silencing the ecdysone synthesis and signaling pathway genes disrupts nymphal development in the whitefly. Insect Biochem. Mol. Biol. *43*, 740–746.

STAR★METHODS

KEY RESOURCES TABLE

REAGENT or RESOURCE	SOURCE	IDENTIFIER
Chemicals, Peptides, and Recombinant Proteins		
Multiplex PCR Plus Kit	QIAGEN	Cat# 206151
Nextera XT DNA library prep kit	Illumina	Cat# FC-131-1024
Bleach	Clorox	N/A
Paraformaldehyde	SIGMA-ALDRICH	P6148; CAS: 30525-89-4
Triton X-100	SIGMA-ALDRICH	X100; CAS: 9002-93-1
Hoechst 33342	Thermo Scientific	Cat# H3570; CAS: 23491-52-3
Deposited Data		
Genome of bacteriocyte sample B1	NCBI SRA	SRA: SRR6148276; https://www.ncbi.nlm.nih.gov/sra/?term=SRR6148276
Genome of bacteriocyte sample B2	NCBI SRA	SRA: SRR6148277; https://www.ncbi.nlm.nih.gov/sra/?term=SRR6148277
Genome of head sample H1	NCBI SRA	SRA: SRR6148278; https://www.ncbi.nlm.nih.gov/sra/?term=SRR6148278
Genome of head sample H2	NCBI SRA	SRA: SRR6148279; https://www.ncbi.nlm.nih.gov/sra/?term=SRR6148279
RNA-Seq data of bacteriocytes of nymph whiteflies	NCBI SRA [4],	SRA: SRR2001505; https://www.ncbi.nlm.nih.gov/sra/?term=SRR2001505
RNA-Seq data of bacteriocytes of adult whiteflies	NCBI SRA [13],	SRA: SRR1523521; https://www.ncbi.nlm.nih.gov/sra/?term=SRR1523521
Experimental Models: Organisms/Strains		
The whitefly *B. tabaci* MEAM1 culture (mtCOI GenBank accession number KM507785)	[4, 13]	N/A
Software and Algorithms		
Genemarker	SoftGenetics	http://www.softgenetics.com/GeneMarker.php
Trimmomatic v 0.35	[35]	http://www.usadellab.org/cms/?page=trimmomatic
BWA-MEM v 0.7.15-r1140	[36]	http://bio-bwa.sourceforge.net
Picard v 2.10.6	http://broadinstitute.github.io/picard/	https://github.com/broadinstitute/picard
SAMtools v 1.3	[37]	http://samtools.sourceforge.net
Freebayes v 0.9.21	[38]	https://github.com/ekg/freebayes
GATK HaplotypeCaller	[39]	https://software.broadinstitute.org/gatk
IQ-TREE v1.5.5	[40]	http://www.iqtree.org
SNPRelate	[41]	http://bioconductor.org/packages/release/bioc/html/SNPRelate.html
STAR	[42]	https://github.com/alexdobin/STAR
Excel	Microsoft	https://www.microsoft.com/en-us
GraphPad Prism 5	GraphPad Software	https://www.graphpad.com/scientific-software/prism

CONTACT FOR REAGENT AND RESOURCE SHARING

Further information and requests for resources and reagents should be directed to and will be fulfilled by the Lead Contact, Angela E. Douglas (aes326@cornell.edu).

EXPERIMENTAL MODEL AND SUBJECT DETAILS

The whitefly *B. tabaci* MEAM1 culture (mtCOI GenBank accession number KM507785) was obtained from poinsettia (*Euphorbia pulcherrima* Willd. Ex Klotzsch) in Ithaca, NY, USA in 1989. The culture was provided by Dr John Sanderson (Cornell University) to the

authors in 2013, and has subsequently been maintained on dwarf cherry tomato (*Solanum lycopersicum* cv. Florida Lanai) in climate-controlled chambers at 27 ± 1°C with a 14 h light:10 h dark regime. The insects are maintained in large cages with at least 1,000 adults per cage, to maintain the genetic diversity.

METHOD DETAILS

Genetic variation of bacteriocytes in the whitefly population

To investigate genetic variation of bacteriocytes in a whitefly population, single pupae were cut from different leaves of multiple tomato plants in different cages and transferred to individual glass tubes (0.4 × 4cm) until they developed to adulthood. The bacteriocytes were dissected with fine pins from 10 replicate adult female whiteflies, each on a separate glass microscope slide at 40 × magnification, and then washed free of contaminating insect tissues with PBS (pH 7.4). The heads were cut off from the female adults of another ten whiteflies, respectively. For collection of each sample (female head, female bacteriocytes and male head), new pins, new slides and new tips were used to prevent cross-contamination of DNA. All the samples were immediately subjected to DNA extraction using the Nonidet-P40-based protocol. All instruments using for dissections and DNA extraction were pre-sterilized. Ten microsatellite loci developed for *B.tabaci* [43, 44] were amplified by PCR multiplexing with QIAGEN Multiplex PCR Plus Kit, using primers listed in Table S1. The PCR products were sent for fragment analysis. The alleles were analyzed using the software Genemarker (SoftGenetics LLC., USA) following the user manual. Finally, the microsatellite profiles of bacteriocytes and heads were compared.

Genetic variation of bacteriocytes over three generations in cross experiments

To examine genetic variation of bacteriocytes over three sexual generations, each newly-emerged unmated adult female and one newly-emerged unmated adult male (prepared as above) were released into a clip-cage that was secured to the abaxial surface of a tomato leaf at the 3-4 true-leaf stage. Females were allowed to oviposit for one week, and then the two adult insects from seven F0 mating pairs (the females were labeled as a-g) were collected for microsatellite determination of heads (both sexes) and bacteriocytes (females only: the numbers of bacteriocytes of adult males are extremely low, too few for microsatellite analysis), as described above, using 5 microsatellite markers as listed in Table S1. The leaf bearing the eggs (generation F1) produced by each F0 cross was excised from the plant and transferred to a 50 mL plastic tube as described previously [45]. Once the F1 insects emerged, they were collected into individual tubes, as for F0. The F1 female offspring were crossed with the F1 male offspring in 10 cross combinations to generate F2 insects as shown in Data S2. This process was repeated to determine the microsatellite profiles of the insects in the F1 crosses and F2. Finally, the microsatellite profiles of bacteriocytes and heads over the three generations were compared.

In parallel, the PCR products in cross experiments C and F over there generations were also Sanger sequenced to confirm the PCR products are from whiteflies or symbionts using primers for five microsatellite markers.

Library construction and genome resequencing

Two female adult whiteflies were randomly collected from different cages of the whitefly culture. Bacteriocytes were isolated from the individual female adult whiteflies, respectively, and washed with PBS. The heads were separated from the same two insects. All the samples were immediately subjected to DNA extraction using the Nonidet-P40-based protocol with the same precautions as described above to avoid contamination, including cross-contamination between samples. The total DNA was measured by Qubit® 3.0 Fluorometer (Thermo Fisher Scientific Inc.). Illumina paired-end libraries were constructed using the Nextera® xt DNA library prep kit, following the manufacturer's instructions (Illumina, San Diego, CA, USA). These libraries were sequenced on the Illumina NextSeq 500 system with the paired-end mode and the read length of 76 bp.

Variant calling

The raw paired-end reads were trimmed for adapters and low quality bases using Trimmomatic v0.35 [35]. Cleaned read pairs were mapped to the whitefly *B. tabaci* MEAM1 genome [12] using BWA-MEM v 0.7.15-r1140 [36], with –M option to mark split alignments as secondary. Alignments with mapping quality $\geq$ 20 were retained and duplicated reads were marked using Picard v 2.10.6 (http://broadinstitute.github.io/picard/). Variants were pre-called using SAMtools v1.3 [37] and Freebayes v0.9.21 [38], respectively with recommended commands: "samtools mpileup -uf genome_file bam_file | bcftools call -Ov -mv" and "freebayes -F 0.2 -C 2 -p 2 -b bam_file -f genome_file." Variants called by the two programs were filtered separately using the criteria: 1) variant quality $\geq$ 30; 2) depth $\geq$ 10 & $\leq$ 300; 3) no significant strand bias; and 4) no missing genotypes. The overlapped sites from the two filtered calls were extracted and used for base quality score recalibration in the final variant calling using GATK HaplotypeCaller [39]. The GATK analysis was performed following the online Best Practices protocol with default parameters (https://software.broadinstitute.org/gatk/best-practices/). Variants called by GATK were filtered using bcftools (http://samtools.github.io/bcftools/) with the criteria: 1) variant quality $\geq$ 30; 2) minimal depth for each sample $\geq$ 2; 3) at least 20 bp away from an InDel; and 4) no missing genotypes. Phylogenic analyses using biallelic SNPs were performed with IQ-TREE v1.5.5 [40]. Best nucleotide substitution model was chosen by model test function in IQ-TREE, and ascertainment bias correction (ASC) was applied for likelihood calculation on SNP data. Finally, the transversion model (TVMe+ASC) and Jukes-Cantor type model (MK+ASC) were used on phylogeny inference for homozygous SNPs and whole SNPs, respectively. The program was run with 1000 bootstrap replicates and both trees were

mid-point rooted. We also used SNPRelate [41] to perform relatedness analysis using identity-by-descent methods, and this generated consistent tree topology.

Expression of telomere maintenance genes in bacteriocytes

Raw RNA-Seq data of bacteriocytes of nymph whiteflies [4] (Acc# SRR1523521) and adult whiteflies [13] (Acc# SRR2001505) were downloaded from NCBI Sequence Read Archive (SRA). Raw RNA-Seq reads were processed using Trimmomatic [35] to remove adaptor and low quality sequences. The cleaned reads were aligned to the whitefly *B. tabaci* MEAM1 genome [12] using STAR [42]. Following alignments, raw counts for telomere maintenance genes were derived and normalized to fragments per kilobase of exon model per million mapped fragments (FPKM).

Observation of bacteriocytes and nuclei during whitefly embryogenesis

Approximately 30 female adults of whiteflies were released into each of 50 clip-cages attached to the leaves and allowed to lay eggs for 1 h and then discarded. Eggs were collected at day 0 (1 h post oviposition), day 4, day 5, day 6, day 7 and day 8 after deposition. Eggs deposited at day 0 were dechorionated by 60% Clorox bleach (3.6% hypochlorite) in PBS for 5 min and then wash with PBS twice, fixed by 4% paraformaldehyde (PFA) at room temperature for 1 h, and then permeabilized with 0.1% Triton X-100 in PBS for 1.5 h. The samples were incubated with Hoechst 33342 (10 $\mu g\ ml^{-1}$ in PBS, Thermo Scientific) at room temperature for 20 min. After the dechorionation treatment, the nuclei in the embryos at late embryogenesis did not stain well. So, we designed a pin-puncture approach to promote permeation of the reagent and dye into eggs. Punctured eggs at days 4-8 after deposition and bacteriocytes dissected from eggs at day 7 after deposition were fixed in 4% PFA at 4°C overnight and then permeabilized with 0.1% Triton X-100 in PBS at room temperature for 2 h. The samples were incubated with Hoechst 33342 (10 $\mu g\ ml^{-1}$ in PBS, Thermo Scientific) in PBS overnight at 4°C. Images were collected and analyzed on a Zeiss LSM 700 confocal microscope. The diameters of the bacteriocytes in eggs and of the nuclei of bacteriocytes and embryo cells were determined by software ZEN for the Zeiss LSM 700 confocal microscope, using five eggs at each stage of embryogenesis and five embryo nuclei per egg. Bacteriocyte and nucleus volume was calculated as $4/3\pi r^3$ as described previously [4].

QUANTIFICATION AND STATISTICAL ANALYSIS

Analysis of volume of bacteriocytes and nuclei

The volume of five embryo nuclei in each egg was averaged as one biological replicate. This value from each of five embryos was used to calculate the overall mean across all the samples. The statistical significance of variation in the volume of bacteriocytes and nuclei was evaluated using ANOVA at a 0.05 level in Microsoft Excel.

DATA AND SOFTWARE AVAILABILITY

The accession numbers for the raw reads of bacteriocyte genomes and head genomes reported in this paper are SRA: SRR6148276 (B1), SRA: SRR6148277 (B2), SRA: SRR6148278 (H1), and SRA: SRR6148279 (H2) (https://www.ncbi.nlm.nih.gov/sra). The accession numbers for the raw reads of bacteriocyte transcriptomes in nymph whiteflies [4] and adult whiteflies [13] reported in this paper are SRA: SRR1523521, SRA: SRR2001505. Other data supporting this study are provided within the paper and Supplemental Information.

Current Biology, Volume *28*

Supplemental Information

Maternal Inheritance of a Single Somatic Animal Cell Displayed by the Bacteriocyte in the Whitefly *Bemisia tabaci*

Junbo Luan, Xuepeng Sun, Zhangjun Fei, and Angela E. Douglas

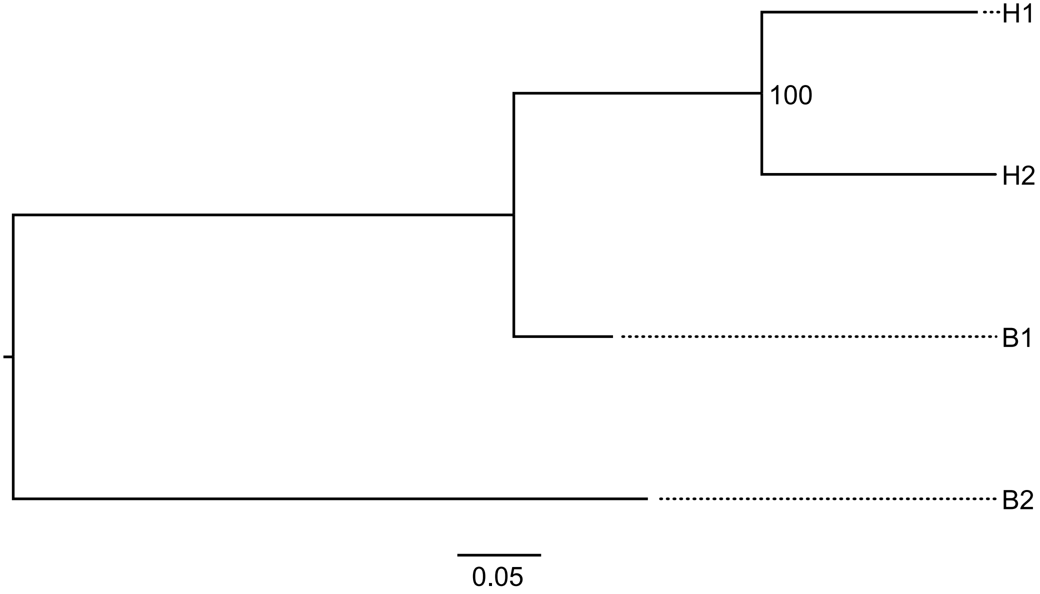

Figure S1. Maximum likelihood phylogeny of bacteriocytes (B1 and B2) and heads (H1 and H2) using all biallelic SNPs (Related to Figure 3 and Table S3). B1 and H1 were isolated from an individual female whitefly, and B2 and H2 from another female whitefly. The two whiteflies were haphazardly collected in the population. The tree is mid-point rooted, and the number on branch is the bootstrap value.

Microsatellite markers	Microsatellite	Primer sequence (5'–3')	Repeat motif	Fluorescent dye color (Applied Biosystems)	Accession numbers	Allele size range (bp) in our lab population	Location in whitefly genome (with the highest identity)
**Population genotyping**	WF1B11	F: GCATTGAACATTTTTCTGCATGCGCG	$(CCTGA)_{12}$ imp	FAM	JQ436838	136-168	Scaffold54198
		R: GCACACAGCTCTCCAAAAGAAAGGTC					
	WF2H06	F: TATTCGCCAATCGATTCCTT	$(TTTG)_{11}$ imp	FAM	JQ436844	172–196	Scaffold684
		R: CGGCGGAAATTTCGATAAA					
	WF2A02	F: GAAGTGCATAAAAACATCGTCGCCG	$(CTTT)_{10}$ imp	PET	JX853746	155	Scaffold1034
		R: CAATTGAAAGGCTAGACTCCCTGGC					
	WF1D04	F: GTTGTTAGGTTACAGGGTTTGTC	$(CAAA)_{16}$	PET	JQ436839	124-168	Scaffold4012
		R: GTCTTTACTTCTCTTTTCCTCCG					
	WF1G03	F: CTCCAAAATGGGACTTGAAC	$(GTTT)_{8}$	NED	JQ436840	158	Scaffold147
		R: GTAGAAGCCACACATACTAGCAC					
	WF2C01	F: ATGATACCGCACGAAAAAGAGGACG	$(GTTT)_{11}$ imp	NED	JQ436842	150-188	Scaffold29488
		R: CTTGAATTACATCAAACGCAGCAGC					
	WF2A05	F: ATTGATCATTTTCGGCTACCTTATC	$(CAAA)_{12}$ imp	VIC	JQ436841	147-155	Scaffold519
		R: CGACCCTTCTGTAAATGATAGACT					
	BtIs1.11	F: ATGTTATGACTATCGCAATC	$(CA)_{8}$	VIC	BV726565	119-133	Scaffold3874
		R: GCAGTGTGTAAGGGTGTC					
	BtIs1.1	F: CCCATAGAACACGCTCC	$(CA)_{7}$	VIC	BV726563	231–261	Scaffold651
		R: CATTGGAAGCCTCGAATAC					
	WF2E11	F: TCTCCAACCATAATTTTTAATCTCG	$(GATT)_{27}$ imp	VIC	JQ436843	244-264	Scaffold130
		R: GTCTGGGCAGGAAAACGAT					
**Cross experiments**	WF2C01	F: ATGATACCGCACGAAAAAGAGGACG	$(GTTT)_{11}$ imp	FAM	JQ436842	150-188	Scaffold29488
		R: CTTGAATTACATCAAACGCAGCAGC					
	WF2H06	F: TATTCGCCAATCGATTCCTT	$(TTTG)_{11}$ imp	PET	JQ436844	172–196	Scaffold684
		R: CGGCGGAAATTTCGATAAA					
	WF1B11	F: GCATTGAACATTTTTCTGCATGCGCG	$(CCTGA)_{12}$ imp	NED	JQ436838	136-168	Scaffold54198
		R: GCACACAGCTCTCCAAAAGAAAGGTC					
	WF1D04	F: GTTGTTAGGTTACAGGGTTTGTC	$(CAAA)_{16}$ imp	VIC	JQ436839	124-168	Scaffold4012
		R: GTCTTTACTTCTCTTTTCCTCCG					
	WF2E11	F: TCTCCAACCATAATTTTTAATCTCG	$(GATT)_{27}$ imp	VIC	JQ436843	244-264	Scaffold130
		R: GTCTGGGCAGGAAAACGAT					

Table S1. Microsatellite loci and microsatellite primers (Related to Figure 1,2 and Data S1,2 and Table S2).

Cross ID	Generations	Markers	Sequences
C	F0	WF2E11	GATTGATTGATTGATTGATTGATTGATTGATTGATTGATTGATTGATTGATTGATTGAATGATTGATTGATTGATTGAATGATTGATTTATTGATTGATTGATTGATTTATTGATTGATTGATGGATCACATCAAGTGAAAATGTGTTTCATTTACCGAAAAAGTGATG
C	F1	WF2E11	GATTGATTGATTGATTGATTGATTGATTGATTGATTGATTGATTGATTGATTGATTGAATGATTGATTGATTGATTGAATGATTGATTTATTGATTGATTGATTGATTTATTGATTGATTGATGGATCACATCAAGTGAAAATGTGTTTCATTTACCGAAAAAGTGATG
C	F2	WF2E11	GATTGATTGATTGATTGATTGATTGATTGATTGATTGATTGATTGATTGATTGATTGAATGATTGATTGATTGATTGAATGATTGATTTATTGATTGATTGATTGATTTATTGATTGATTGATGGATCACATCAAGTGAAAATGTGTTTCATTTACCGAAAAAGTGATG
F	F0	WF2E11	GATTGATTGATTGATTGATTGATTGATTGATTGATTGATTGATTGATTGATTGATTGAATGATTGATTGATTGATTGAATGATTGATTTATTGATTGATTGATTGATTTATTGATTGATTGATGGATCACATCAAGTGAAAATGTGTTTCATTTACCGAAAAAGNGATG
F	F1	WF2E11	GATTGATTGATTGATTGATTGATTGATTGATTGATTGATTGATTGATTGATTGATTGAATGATTGATTGATTGATTGAATGATTGATTTATTGATTGATTGATTGATTTATTGATTGATTGATGGATCACATCAAGTGAAAATGTGTTTCATTTACCGAAAAAGCGATG
F	F2	WF2E11	GATTGATTGATTGATTGATTGATTGATTGATTGATTGATTGATTGATTGATTGATTGAATGATTGATTGATTGATTGAATGATTGATTTATTGATTGATTGATTGATTTATTGATTGATTGATGGATCACATCAAGTGAAAATGTGTTTCATTTTACCGAAAAAGTGATG
C	F0	WF1B11	CTTGACCTGACCTGACCTGACCTGACCTGATCTGACCTGACCTGACCTGACCTGACCTGACCTTT
C	F1	WF1B11	CTTGACCTGACCTGACCTGACCTGACCTGATCTGACCTGACCTGACCTGACCTGACCTGACCTTT
C	F2	WF1B11	CTTGACCTGACCTGACCTGACCTGACCTGATCTGACCTGACCTGACCTGACCTGACCTGACCTTT
F	F0	WF1B11	CTTGACCTGACCTGACCTGACCTGACCTGATCTGACCTGACCTGACCTGACCTGANCTGACCTTT
F	F1	WF1B11	CTTGACCTGACCTGGACCTGACCTGACCTGATCTGACCTGACCTGACCTGACCTGACCTGACCTTT
F	F2	WF1B11	CTTGACCTGACCTGACCTGACCTGACCTGATCTGACCTGACCTGACCTGACCTGACCTGACCTTT
C	F0	WF2H06	TTTTATCAGTCTCATTTTATCAAAACGCTCACCGGCTATTTGTTTGTTTGTTTGNTTGTTTGTTTGTTTGTTTGTTTGT
C	F1	WF2H06	TTTTATCAGTCTCATTTTATCAAAACGCTCACCGCTATTTGTTTGTTTGTTTGTTTGTTTGTTTGTTTGTTTGTTTGT
C	F2	WF2H06	TTTTATCAGTCTCATTTTATCAAAACGCTCACCGCTATTTGTTTGTTTGTTTGTTTGTTTGTTTGTTTGTTTGTTTGT
F	F0	WF2H06	TTTTATCAGTCTCATTTTATCAAAACGCTCACCGCTATTTGTTTGTTTGTTTGTTTGTTTGTTTGTTTGTTTGTTTGT
F	F1	WF2H06	TTTTATCAGTCTCATTTTATCAAAACGCTCACCGCTATTTGTTTGTTTGTTTGTTTGTTTGTTTGTTTGTTTGTTTGT
F	F2	WF2H06	TTTTATCAGTCTCATTTTATCAAAACGCTCACCGCTATTTGTTTGTTTGTTTGTTTGTTTGTTTGTTTGTTTGTTTGT
C	F0	WF2C01	GTTTGTTTGTTTGTTTGTTTGTTTGTTTGTTGGTTTGTTTGTTTGTTTGTTT
C	F1	WF2C01	GTTTGTTTGTTTGTTCGTTNGTTTGTTTGTTTGTTTGTTTGTTTGTTTGTTT
C	F2	WF2C01	GGTTGTTTGTTTGTTTGTTTGTTTGTTTGTTTGTTTGTTTGTTTGTTTGTTT
F	F0	WF2C01	GTTTGTTTGTTTGTTTGTTTGTTTGTTTGTTTGTTTGTTTGTTTGTTTGTTT
F	F1	WF2C01	GTTTGTTTGTTTGTTTGTTTGTTTGTTTGTTTGTTTGTTTGTTTGTTTGTTT
F	F2	WF2C01	GGTTGTTTGTTTGTTTGTTTGTTTGTTTGTTTGTTTGTTTGTTTGTTTGTTT
C	F0	WF1D04	NAAACAAACAAACAAACAAACAAACAAACAAACAAACAAACAAACAAA
C	F1	WF1D04	CAAACAAACAAACAAACAAACAAACAAACAAACAAACAAACAAACAAA
C	F2	WF1D04	CAAACAAACAAACAAACAAACAAACAAACAAACAAACAAACAAACAAA
F	F0	WF1D04	CAAACAAACAAACAAACAAACAAACAAACAAACAAACAAACAAACAAA
F	F1	WF1D04	CAAACAAACAAACAAACAAACAAACAAACAAACAAACAAACAAACAAA
F	F2	WF1D04	CAAACAAACAAACAAACAAACAAACAAACAAACAAACAAACAAACAAA

Table S2. Sequences of microsatellite loci derived by Sanger sequencing of PCR products of bacteriocytes isolated from adult female whiteflies in cross experiments C and F over three generations (Related to Figure 2 and Data S2).

	H1	H2	B1	B2
Raw reads				
#Read pairs	204,888,779	92,341,548	92,242,175	68,994,911
Total nucleotides (bp)	31,143,094,408	14,035,915,296	14,020,810,600	10,487,226,472
Cleaned & non-duplicated reads				
#Read pairs	55,144,510	38,543,693	23,046,421	48,724,623
Total nucleotides (bp)	8,265,564,207	5,784,811,536	3,459,552,051	7,322,190,441
Mean coverage	12.0×	8.4×	5.0×	10.6×
#SNPs	453,208			
#Biallelic	451,709			
#Homozygous	134,380	165,575	162,822	296,728
#Heterozygous	317,329	286,134	288,887	154,981
#Multiallelic	1,499			
#INDELs	60,348			

Table S3. Summary of genome resequencing and SNPs (Related to Figure 3).

Bacteriocyte B1 and head H1 are from one individual whitefly, and bacteriocyte B2 and head H2 from another individual whitefly.

Genes	Whitefly genome ID	NB_FPKM	AB_FPKM
Telomerase reverse transcriptase (TERT1)	Bta11058	1.00	1.41
Telomerase reverse transcriptase (TERT2)	Bta11059	2.37	2.62
Telomerase Cajal body protein 1 (TCAB1)	Bta15778	4.37	5.54
Telomerase-binding protein EST1A	Bta15441	15.34	15.75
Telomere length regulation protein TEL2	Bta09854	12.98	19.41

Table S4. Expression of telomere maintenance genes in whitefly bacteriocytes of both nymphs and adults (Related to Figure 3).

NB denotes bacteriocytes of nymph whiteflies and AB denotes bacteriocytes of adult whiteflies.

The data were obtained by mapping raw reads of bacteriocyte transcriptome [S1, S2] to whitefly genome [S3] and the FPKM value (fragments per kilobase of transcript per million fragments mapped) was calculated.

Supplemental References:

S1 Luan, J.B., Shan, H.W., Isermann, P., Huang, J.H., Lammerding, J., Liu, S.S., and Douglas, A.E. (2016). Cellular and molecular remodelling of a host cell for vertical transmission of bacterial symbionts. Proc. R. Soc. Lond. B. Biol. Sci. *283*, 20160580.

S2 Luan, J.B., Chen, W., Hasegawa, D.K., Simmons, A.M., Wintermantel, W.M., Ling, K.S., Fei, Z., Liu, S.S., and Douglas, A.E. (2015). Metabolic coevolution in the bacterial symbiosis of whiteflies and related plant sap-feeding insects. Genome Biol. Evol. *7*, 2635-2647.

S3 Chen, W., Hasegawa, D.K., Kaur, N., Kliot, A., Pinheiro, P.V., Luan, J., Stensmyr, M.C., Zheng, Y., Liu, W., Sun, H., et al. (2016). The draft genome of whitefly *Bemisia tabaci* MEAM1, a global crop pest, provides novel insights into virus transmission, host adaptation, and insecticide resistance. BMC Biol. *14*, 110.
